# The contribution of a central pattern generator in a reflex-based neuromuscular model

**DOI:** 10.3389/fnhum.2014.00371

**Published:** 2014-06-26

**Authors:** Florin Dzeladini, Jesse van den Kieboom, Auke Ijspeert

**Affiliations:** BioRob, School of Engineering, Institute of Bioengineering, École Polytechnique Fédérale de LausanneLausanne, Switzerland

**Keywords:** feedback, CPG, humans, locomotion, models, biological, movement, periodicity

## Abstract

Although the concept of central pattern generators (CPGs) controlling locomotion in vertebrates is widely accepted, the presence of specialized CPGs in human locomotion is still a matter of debate. An interesting numerical model developed in the 90s’ demonstrated the important role CPGs could play in human locomotion, both in terms of stability against perturbations, and in terms of speed control. Recently, a reflex-based neuro-musculo-skeletal model has been proposed, showing a level of stability to perturbations similar to the previous model, without any CPG components. Although exhibiting striking similarities with human gaits, the lack of CPG makes the control of speed/step length in the model difficult. In this paper, we hypothesize that a CPG component will offer a meaningful way of controlling the locomotion speed. After introducing the CPG component in the reflex model, and taking advantage of the resulting properties, a simple model for gait modulation is presented. The results highlight the advantages of a CPG as feedforward component in terms of gait modulation.

## 1. Introduction

Central pattern generators (CPGs) are networks of neural cells that can generate coordinated rhythmic patterns in the absence of sensory feedbacks. The idea that CPG control locomotion in lower vertebrates has been widely accepted for several decades (Grillner and Wallen, 1985). Although many observations tend to favor the presence of such components in higher vertebrates (see MacKay-Lyons, 2002 for a review), the presence of specialized CPGs in human locomotion is still a matter of debate (Dimitrijevic et al., 1998). An interesting numerical model developed by Gentaro Taga in the 90s’ demonstrated the role that CPGs could play in human locomotion. It was shown that walking and running could emerge from a rhythmic interaction (modeled by coupled oscillators, i.e., CPGs), between the central nervous system, the musculo-skeletal-system and the environment. The CPGs were modeled as a network of oscillators, coupled with the environment through joint angles and ground reaction forces (Taga, 1994). The intriguing robustness of the generated gaits against mechanical perturbations and changes in the environment was attributed to the use of CPGs and feedbacks, respectively, highlighting the important role of both components. However, more recently, a neuro-musculo-skeletal model (denoted FBL, for Feedback Based Locomotion) solely driven by reflex loops was proposed by Geyer and Herr (2010). The model showed a stability to perturbations similar to the previous model, without any CPG components, questioning the conclusions drawn by Taga et al. regarding the importance of CPGs to resist perturbations. Furthermore, the properties of the gaits produced by the FBL model were—in terms of muscles activity, joints angles and torques patterns—surprisingly close to those observed in humans. Yet, an important feature the reflex-driven neuro-musculo-skeletal system was unable to reproduce was the control of speed. Indeed, while in Taga’s model, speed was controlled by a simple unique variable (the frequency of the oscillators), such a strategy is inapplicable in the reflex model. Although a preliminary speed control strategy has been proposed by Song and Geyer (2012), its complexity compared to the very simple descending signals, originating from the brain stem, able to control locomotion (found in lower vertebrates, such as the lamprey and the salamander, and even in cats) makes their relevance, from a biological point of view, questionable.

Given the striking properties of the reflex model, we wanted to study the possible benefits that a CPG would add to the model. We hypothesized that the reflex model would benefit from the presence of CPGs in terms of gait speed/step length control. The CPG component is derived from the feedback pathways, following an idea from Kuo (2002), where CPGs are viewed as feedback predictors. We use a variety of models combining CPG and feedbacks in different ways to study the relative importance of the different feedbacks/feedforward pathways. Finally, taking advantage of the properties of the CPG, a simple model for speed modulation is presented.

## 2. Materials and methods

In this section, we describe step-by-step how we generate the CPG-based extension of Geyer’s FBL model, referred to as 3FBL (for FeedForward and Feedback Based Locomotion). We first present our implementation of the FBL model and detail its optimization. This model demonstrates that simple delayed feedback loops (i.e., delayed linear mapping between sensors state and muscles activities) combined with a simplified musculoskeletal model (lower limb model of human based on anthropometric data, actuated by seven Hill muscle models per limb) is sufficient to generate walking at various frequencies and step lengths. Furthermore, when the objective function used for the optimization process includes a metabolic cost minimization criterion, the generated angles, torques and muscles activation are comparable to human walking data (replicating results found in Geyer and Herr, 2010 and Wang et al., 2012). Despite the interesting properties of the model, an important limitation is that, once a walking gait at a given speed and step length is obtained, the only way to modulate it is by tuning of the multiple feedback gains. For example, in Song and Geyer (2012), a speed controller has been derived based on feedback gains tuning. The proposed controller is able to switch between gaits of different speeds, but the strategy remains complex. In short, speed changes are obtained by switching between different sets of feedback gains; increasing speed is done by (1) switching to a set of gains that generate an acceleration, and (2) once the desired speed is reached, switching to a set of gains that generate a gait of the desired speed.

The gait modulation strategy we propose is based on evidence from lower vertebrates and quadrupeds suggesting that simple low dimensional descending signals are enough to modulate walking (speed changes and gait transitions) (Grillner and Wallen, 1985). Our strategy to introduce CPGs as a feedforward component is based on the assumption that CPGs can be viewed as feedback predictors. In other words, CPGs should be able to reproduce any feedback signals generated by a stable walking gait of the FBL model. Since the feedback signals can be of any shape, we do not want to make strong assumptions on the class of pattern. Therefore, we will use a special class of oscillators called “morphed non-linear phase oscillators,” that have the ability to generate limit cycles of arbitrary shape (Ajallooeian et al., 2013). Note that we do not model individual neurons but rather use an abstract model of biological CPGs represented as a dynamical system exhibiting limit cycle behavior. This strategy is commonly used to test hypothesis on the role of biological CPGs (Ijspeert, 2008).

The CPGs will then be combined with feedback pathways using the strategy presented in Kuo (2002), offering an elegant and easy way to study the relative importance of the different feedback pathways. The proposed strategy will also permit to highlight the pathways that can be used as speed and step length modulators.

### 2.1. FBL description

The pure feedback-based neuromuscular model of human locomotion (or FBL model) refers to a bio-inspired neuromuscular bipedal walking model developed by Geyer and Herr (2010) that we reimplemented and use as a starting point for our study. The following description is thus largely inspired by their work. Any differences with the original model will be explicitly stated.

In this study, all experiments are done using an implementation of the NMM library (a freely accessible C++ library that we developed to simulate neuromuscular models^1^ on the Webots robotic environment platform (Michel, 2004). This webots implementation^2^ is based on an anthropometric model of human lower body (see Supplementary Figure 3, anthropometric data from Winter, 2009).

**Figure 1:**
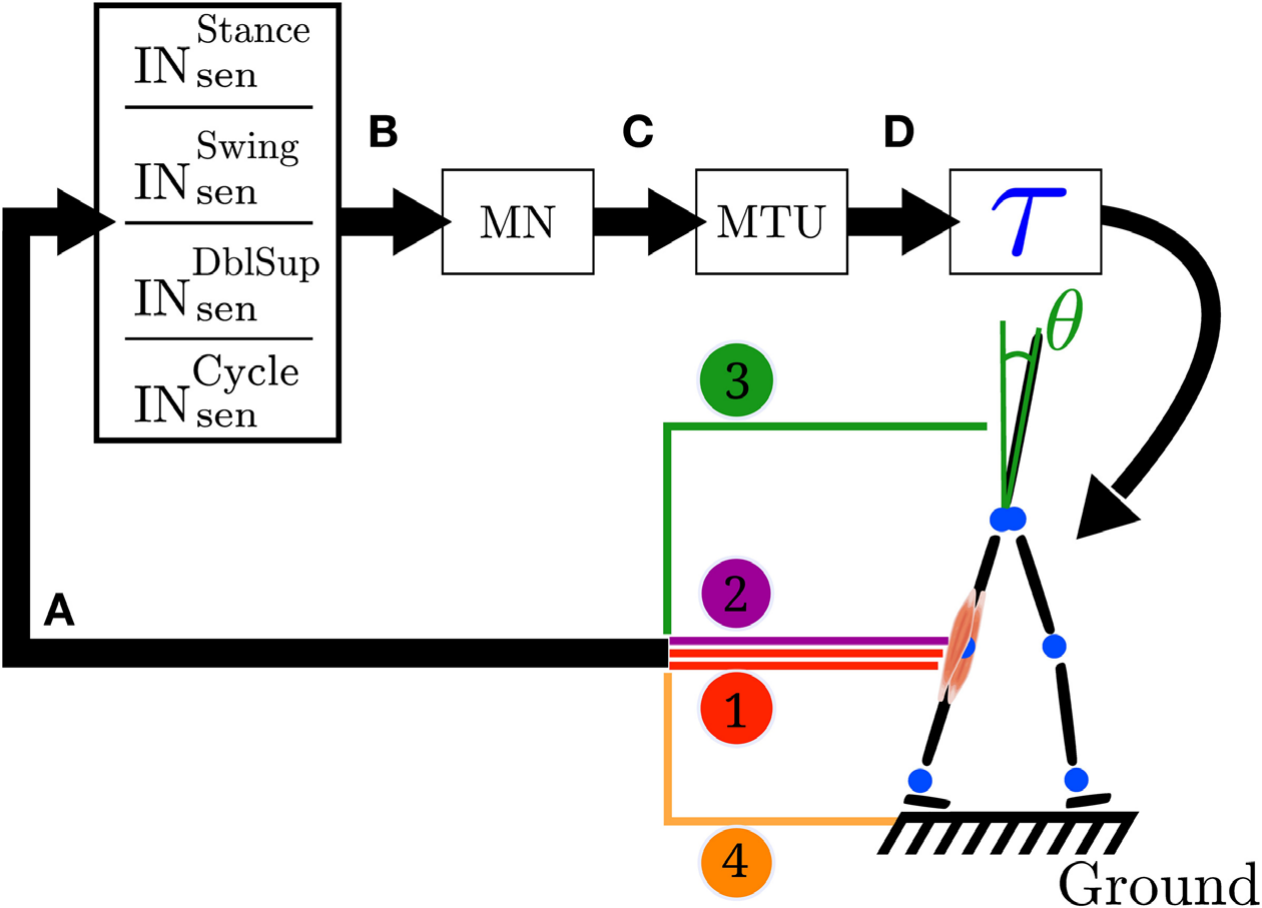
**Closed loop information flow of the FBL model**. **(A)** Sensors signals stimulate (see Equation 1) a set of sensory interneurons (IN_sen_). The sensors signals are represented by the colored line; 1 represents the muscle sensors, 2 represents the joint overextension/flexion prevention sensors, 3 represents the stability sensor generating a signal to maintain the trunk upright and 4 represents the ground sensors. There are four different types of sensory interneurons: IN^stance^_sen_ which are active only during stance, IN^swing^_sen_ only during swing, IN^dblsup^_sen_, only during the double support phase and IN^cycle^_sen_ during the whole cycle. **(B)** Each IN_sen_ is connected to a unique motoneuron (MN). However a given MN receives inputs from several IN_sen_. Connections between IN_sen_ and MN follow Equation 2. **(C)** In turn, each MN stimulates its corresponding muscle tendon unit (MTU). **(D)** Each MTU contributes to a torque (τ) on one or two joints, depending on whether it models a uni- or bi-articular muscle. Finally, the action of all the muscles on the body generates a movement, which induces a change in the sensors state and thereby closes the loop. Note that in the original model the link between sensors states and muscles activities is direct (i.e., no intermediary stage), while here the sensors to muscles mapping is separated in three more biologically relevant stages: sensory interneurons (IN_sen_), motoneurons (MN) and muscle tendon units (MTU). Note that both the original and the FBL model are computationally equivalent.

The FBL model uses feedback rules connecting different sources of sensory information (comprising muscle force and length feedbacks, ground reaction forces and joint angles) to Hill-type muscle models (details concerning the muscle model can be found in Geyer et al., 2003), which in turn generate effective joints torques. A state machine is used to switch between two sets of feedback rules: one to generate the stance phase control (mainly extensor muscles activity) and one to generate the swing phase control (mainly flexor muscles activity). Ground sensors placed under the feet are used to detect the state transition (takeoff and touchdown). The generation of the gait cycle is done through reflexes represented by a sequence of time delayed reactions (see Figure 1).

While in the original model the link between sensors states and muscles activities was direct (i.e., no intermediate stage), in our work we separate the sensors to muscles mapping in three more biologically relevant stages (see Figure 1 for details): sensory interneurons (IN_sen_), motoneurons (MN) and muscle tendon units (MTU). The intermediate stages are added in order to prepare the extension of the model and makes no functional differences with the original model, as long as the overall delay between sensors and muscle activities is identical in both models. Stages A to C are implemented using the connection model defined in section 2.2.4. The sensors to torque mapping noted A to D (schematically represented in Figure 1) are presented below (see Supplementary Table 3 for a description of the different vector/matrices used):

- Sensors to Interneurons
  The activity of all interneurons can be written, in matrix form as:
  (1) $$X_{{\text{in}}_{\text{sen}}}=min\;{\left\{1,\;max\;\{0,\;W{\tilde{X}}_{\text{sen}}\;\}\right\}}^{T}$$
  Where *X*_in_sen__ is a vector of sensory interneurons activities, $\tilde{X}$_sen_ is a vector of delayed sensors activities. *W* is the connection weights matrix linking the sensors and the interneurons. Table 1 gives the list of the sensory interneurons present in a given limb.
- Interneurons to Motoneurons
  Given limbs state *s* = (*S*_left_, *S*_right_) (with *S*_left_, *S*_right_ ∈ *S* = {ST, STend, SW}, where ST, SW and STend stand for stance, swing and double support finishing stance respectively) the activity of all the motoneurons can be written, in matrix form as:
  (2) $$X_{\text{mn}}=G^{s}X_{{\text{in}}_{\text{sen}}}+X_{\text{mn}}^{0}$$
  Where: *X*_mn_ is the vector of motoneurons activities acting on limb *L*, *X*_in_sen__ is a vector of sensory interneurons activities, in this case we assume no delay between interneurons and motoneurons (i.e., $\tilde{X}$_in_ = X_in_). *X*^0^_mn_ is a vector of basal motoneurons activities. *G^s^* is a boolean matrix representing the connection state from interneurons to motoneurons given a limb state *s*. It ensures that the interneurons act on the motoneurons only when needed (i.e., stance feedback loops are active only during stance, swing feedback loops only during swing). For example if the interneuron *i* = 18 is connected to a motoneuron *j* = 3 and active only during left swing then *G^s^*(3, 18) = 1 if *s* = (SW,·). Given a limb state *s*, the state of the considered limb *S*_limb_, where limb can be either left or right is defined as a function of the level of the vertical ground reaction forces *GRF*^y^_limb_ and the state of the contralateral limb *S*_contra_. When *GRF*^y^_limb_ < 0.1, the limb is considered in swing (*S*_limb_ = SW). If *GRF*^y^_limb_ ⩽ 0.1 and *S*_contra_ switches from SW to ST then the current limb is in finishing stance (*S* = STend) otherwise the limb is in stance (*S*_limb_ = ST).
- Motoneurons to muscle activities
  A motoneuron acts on only one MTU, consequently the equation linking motoneurons to the MTUs stimulation is simply given by:
  (3) $$X_{\text{mtu}}={\tilde{X}}_{\text{mn}}$$
  Where: *X*_mtu_ is a vector of MTUs stimulation and $\tilde{X}$_mn_ is a vector of delayed motoneurons activities. The MTU stimulation is constrained to the [0.01, 1] interval. The lower bound of 0.01 is there to model the muscle tone (i.e., a minimal level of tension always produced by the motoneurons inervating a muscle). Its purpose is to permit quicker recruitment of muscles by maintaining a minimal non-zero level of tension. The MTU activation level *A* constrained to the [0, 1] interval is linked to the MTU stimulation level by a first order differential equation modeling the excitation-contraction coupling:
  (4) $$\frac{dA}{dt}=\tau_{A}(X_{\text{mtu}}-A),\tau_{A}=100[s^{-1}]$$
- Muscle activities to joint torques
  The overall torque τ_j_ acting on joint *j* is given by:
  $$\tau_{j}\;=\sum_{m\epsilon j}\tau_{m,j}+\tau_{j}^{lig}$$
  Where τ*^lig^_j_* is the torque generated by the ligaments of joint *j*, τ*_m, j_* = *F_m_* · *r_m_(ϕ_j_*) is the torque generated by a MTU *m* on joint *j*, *F_m_* is its force and *r_m_* is the moment arm between MTU *m* and joint *j* (constant *r*_0_ for hip joints and *r*_0_*cos*(ϕ − ϕ_max_) for knee and ankle joints, the *r*_0_ and ϕ_max_ values associated to each muscle-joint couples are given in Table 2).

**Table 1:** **List of the FBL sensory interneurons**.

**Sensory interneurons**				
**Abbreviation**	**Type**	**From**	**To**	**ACTIVE_DURING**
GAS←GAS MFF, ST	1b	GAS	GAS	Stance
GLU←GLU MFF, SW	1b	GLU	GLU	Swing
HAM←HAM MFF, SW	1b	HAM	HAM	Swing
SOL←SOL MFF, ST	1b	SOL	SOL	Stance
TA←SOL MFF, ST	1b	SOL	TA	Stance (−)
VAS←VAS MFF, ST	1b	VAS	VAS	Stance
TA←TA MLF CY	1a	TA	TA	Cycle
HF←HAM MLF SW	1a	HAM	HF	Swing (−)
HF←HF MLF SW	1a	HF	HF	Swing (−)
HF←GSIF ST	3, 4	iFoot, Trunk	HF	Stance
HAM←GSIF ST	3, 4	iFoot, Trunk	HAM	Stance
GLU←GSIF ST	3, 4	iFoot, Trunk	GLU	Stance
VAS←GCF STend	4	cFoot	VAS	Stance end (−)
HF←TLF SW	3	Trunk	HF	Swing
VAS←KNEE OPF	2	KNEE	VAS	Angle off (−)

*The first column gives the abreviation of the interneuron. The abreviation indicates from which sensor the interneuron receives input from and to which MN it sends its output and is constructed as follow: MN←INsen_TYPE, ACTIVE_DURING. MN represents the motoneuron onto which the interneuron acts. If not specified, the motoneuron onto which the interneuron acts is on the same side as the sensors side (i.e., ipsilateral). INsen_TYPE represents the interneuron type. There are six different sensory interneurons; MFF (MTU force feedback), MLF (MTU length feedback), GSIF (ground and stability ipsilateral feedback), GCF (ground contralateral feedback), OPF (overextension prevention feedback), TLF (trunk lean feedback). ACTIVE_DURING indicates when the feedback is active; ST: feedback is active during stance, STend: feedback is active during double support finishing stance, SW: feedback is active during swing, CY: feedback is active during the whole cycle, AO: the feedback is active only when the angle of the corresponding joint goes beyond a certain limit, this is used only for the knee joint where the limit is fixed and set to 170°. The second column gives the type of the interneuron, as described in section 2.2.3. The third and fourth columns indicate the start and target of each feedback pathway. The last column specifies in which part of the cycle the feedback is active, the (−) sign refers to a inhibitory effect*.

**Table 2:** **List of the seven different muscles used in the FBL and derived models: GLU for gluteus, HF for hip flexor, VAS for vasilus, GAS for gastrocnemius, TA for tibialis, HAM for hamstring and SOL for soleus**.

**MTUs list and joints related parameters**				
	**Action**	**r_0_[m]**	**φ_max_[deg]**	**φ_ref_[deg]**
GLU	hip ext.	0.1	–	150
HF	hip flex.	0.1	–	180
VAS	knee ext.	0.06	165	125
SOL	ankle ext.	0.05	110	80
TA	ankle flex.	0.04	80	110
HAM	hip ext. knee flex.	0.08	−, 180	155, 180
GAS	ankle ext. knee flex.	0.05	110, 140	80, 165

*The last two rows (HAM and GAS muscles) corresponds to bi-articular muscles (i.e., they span two joints), other rows are for uni-articular muscles. The second column shows the resulting action on the joint(s) onto which the muscle acts. The third column corresponds to the lever arm used for torque calculation. The fourth column gives the angle at which the action of the muscle on the joint is maximum (absent for the hip joint). The last column gives the reference angle of the muscle (i.e., the angle that corresponds to the muscle rest length)*.

### 2.2. FBL components

#### 2.2.1. Ligament model

In animals, a ligament forms the joint that maintains two bones together. It also ensures that the angle formed by the bones stays within a given range. Its action is against the movement and engages only when the angle is beyond a certain limit, which depends on the joints (see Supplementary Table 5). Ligaments are modeled as non-linear spring damper acting as soft limit on the joints (Geyer and Herr, 2010). When the angle goes beyond the limit of the joint and the angular speed is not big enough to bring back the joint in its normal range a force is generated. The resulting torque τ*^lig^_j_* acting on joint *j* is modeled as:

(5) $$\tau^{lig}=\{\begin{array}{ll} k\cdot \Delta \phi \;\cdot \;\left(1-\omega /\omega_{ref}\right) & \text{ if }\Delta \phi >0,\;\omega /\omega_{ref}>-1\\ 0 & \text{ else } \end{array}$$

Where *k* = 17.19 [*Nm/rad*] is the spring damper stiffness, ω*_ref_* = 1.74·10^−2^ [*rad/s*] is the reference angular speed, used to normalize the joint angular speed, Δϕ is the angle by which the joint limit is exceeded (i.e., difference between the actual angle and the limit angle, the axes are chosen so that Δϕ > 0 when the joint limit is passed) and ω [*rad*^−1^] is the angular speed (the axes of rotation are chosen so that ω > 0 when the angle is going toward the joint limit angle).

Note that this model of non-linear spring damper is also used in the model of H. Geyer to model the ground reaction forces to foot contacts. Here the contact of the robot with the ground are managed by the physical simulator of Webots.

#### 2.2.2. Muscle model

The muscle model is based on the Hill model (Hill, 1938) and was developed by Geyer et al. (2003). A muscle is modeled together with its respective tendon (called muscle tendon unit, or MTU). An active, contractile element (CE) with two passive parallel elements (buffer elasticity BE and parallel elasticity PE) form the muscle, see Supplementary Figure 4. The active element represents the muscle active contractile element, while the two passive elements model the physical properties of the muscle fibers. The BE element prevents the muscle from collapsing, while the PE prevents the muscle length from going beyond a certain length. The tendon is modeled as a passive element in series with the muscle, called series elasticity (SE). The full mathematical formulation can be found in Geyer et al. (2003). The signal sent to the muscle by the motoneuron is related to the activity of the muscle with a first order differential equation accounting for neural delays, see section 2.2.4.

The force of a specific muscle *j* is linked to its activation level *A_j_* by:

(6) $$F_{\text{CE}}=F_{\text{max}}\cdot f_{l}(l_{\text{CE}})\cdot f_{v}(v_{\text{CE}})\cdot A_{j}$$

Where : *F*_CE_ is the muscle force, *F*_max_ is the maximum force generated by the muscle, *f_l_* and *f_v_* respectively models the length-force and velocity-force relationship capturing main biological features of muscles, *f_l_* and *f_v_* equation can be found in Geyer et al. (2003). Given the muscle diagram depicted in **Figure 4** and applying Newton’s third law of motion, we have that the net force generated by the muscle tendon unit (*F_m_*) equals the force of the tendon *F*_SE_:

(7) $$F_{m}=F_{\text{SE}}=F_{\text{CE}}+F_{\text{PE}}-F_{\text{BE}}$$

The only unknown variables are the length and speed of the contractile element from which all muscle variables can be derived. Details on how *v*_CE_ is calculated can be found in Geyer et al. (2003). *l*_CE_ is then derived by integrating *v*_CE_.

#### 2.2.3. Sensors model

There are four different types of sensors (see Figure 1).

- Muscle sensors (type 1): there are two muscle sensors types. (1a) muscle length sensors, modeling the secondary muscle spindles and (1b) muscle force sensors, modeling the Golgi tendons.
- Joint overextension/flexion prevention sensor (type 2): its intensity is proportional to the difference between the maximum tolerated angles and actual joint angle, and its direction is always against the movement. It is used to prevent knee joint overextension.
- Ground sensor (type 3): as in the original model (Geyer and Herr, 2010), there are two sensors under each foot that feel the reaction forces of the ground, located at the toe and heel position. In our case, the heel and toe sensors are provided by a Webots module called a TouchSensor that returns the cumulative force currently exerted on the sensor’s body. Then, as in the original model, the value returned by the ground sensor is defined as being equal to the sum of the toe and heel sensors normalized by the total weight of the model.
- Stability sensor (type 4) measures the angle of the trunk in world coordinate and is used by stability feedback to bring the trunk toward a reference angle. These feedbacks are proportional-derivative control adapted to act on muscles and can be viewed as abstract models of descending pathways responsible for balance control originating from the cerebellum and the vestibular system.

#### 2.2.4. Connection model

In the FBL, walking is generated by a sequence of time delayed reactions (or feedback loops) that connect sensory interneurons to muscles stimulation. The state of the output (*y_j_*) is modeled as an affine transform of the sum of delayed weighted inputs ($\tilde{x}$*_i_* = *x_i_(t − T_i, j_*)):

(8) $$\begin{array}{l} y_{j}=f\left(W^{\prime }\tilde{X}\right)=f\left(\sum_{i\varepsilon \;Input}\left(w_{j,i}{\tilde{x}}_{i,j}\right)\right)\\ \;=min\left\{1,\;max\left\{0,\sum_{i\varepsilon \;Input}\left(w_{j,i}\cdot x_{i}\left(t-T_{i,j}\right)\right)+x_{j}^{0}\right\}\right\} \end{array}$$

Where the i-th index refers to input *i* and j-th index refers to the output *j*. Input-Output pairs are sensory neurons-sensory interneurons (stage A), sensory interneurons-motoneurons (stage B) and motoneurons to MTUs stimulation (stage C) shown on Figure 1. $\tilde{x}$*_i, j_* represent delayed input neuron activities meaning that a change in an input neuron will not affect the output neuron instantaneously but does so after a delay *T_i, j_* (modeling the fact that traveling speed of spikes depend on the properties of the nerve fiber). The delays are estimated assuming an average nerve fiber conductance of 80 m/s and estimated length between sensors and spinal cord. Note that the conductance of 80 m/s is the lower bound of extrafusal muscle fibers, golgi tendon organ and muscle spindle Ia conduction velocity (Siegel et al., 2006). We use three differents delays. A 2.5 ms delay to model the delay from hip muscles sensors and trunk stability sensors to their corresponding sensory interneuron and from the hip motoneurons to hip muscles. A 5 ms delay to model the delay from knee muscles sensors and knee joint angles sensors to their corresponding sensory interneurons and from the knee motoneurons to knee muscles and finally. A 10 ms delay for the ankle muscles sensors and ground sensors to their corresponding sensory interneuron and from the ankle motoneurons to ankle muscles. We assume no delay between sensory interneurons and motoneurons. *w_j, i_* is the connection weight from input *x_i_* to output *y_j_* and *x*^0^_*j*_ is the basal activity of the output (in vector format *W* is the vector of weights and $\tilde{X}$ is the vector of delayed input activity). The output is always constrained to the [0, 1] interval. For a neuron it can be viewed as its normalized firing frequency (1 meaning the neuron is firing at its maximum rate and 0 the neuron is not firing at all), for an MTU it can be viewed as a percentage of maximum muscle stimulation.

### 2.3. FBL simulation environment and optimization

The model is implemented as described in Geyer et al. (2003) and Geyer and Herr (2010), i.e., 6° of freedom all constrained to the sagittal plane and 7 Hill type based muscles per limb. Simulations run with a time step of 1 ms. All differential equations are solved with a fourth order RungeKutta method, except for the muscle velocity which is integrated using the Euler method (as described in Geyer et al., 2003). In order to ensure convergence of the integration process, the integration time step of the muscle is reduced by a factor of 20 in comparison to the simulation time step.

Concerning the optimization, the open parameters of the system are the motoneurons basal activities (*X*^0^_mn_ in Equation 2), the sensors parameters (trunk reference angle of the stability feedback, muscle length feedback offsets) and the feedback gains (non-zero values of matrix *W_in, sen_* in Equation 1). The full model has 25 open parameters (the parameters and their associated ranges are given in Supplementary Table 1). In Geyer and Herr (2010), the parameters values were hand-tuned. When using those parameter values in our implementation, the produced gait shows a velocity of 1.1 [m/s]. The generated angles have a correlation with human data of 0.6, 0.7, and 0.9 for the HIP, KNEE, and ANKLE joint, respectively. The differences in produced gait between the original Geyer model and our implementation (for a given set of parameters) can be explained by the fact that we use a different simulation environment, bringing differences in the contact model and ground sensors. In almost all subsequent articles on FBL enhancement, optimization algorithms are used to set the parameters values. For example, in Song and Geyer (2012), the parameters were optimized to generate gaits of different speeds. The parameters were then analyzed in order to study the possibility to generate a speed controller through the direct modulation of reflex gains. The objective function used took into account the difference between target velocity and current velocity, a penalty term accounting for knee overextension and an energy expenditure term based on Bhargava et al. (2004).

In this article we also use optimization to instantiate parameters values of the FBL model. Since at least two criteria are always used (i.e., the minimization of energy and the penalty term accounting for knee overextension, and more as soon as one wants to optimize for an extra parameter, such as speed or step length), a good handling of multi-criteria evaluation is mandatory. We use a lexicographic ordering extension on top of the PSO (Particle Swarm Optimization Kennedy and Eberhart, 1995) algorithm to handle multi-objectives fitness functions. Lexicographic ordering can be used only if the objectives can be written as constraints and ensures that the multi-objective optimization remains on the Pareto Front (Czyzżak and Jaszkiewicz, 1998; Li et al., 2008). Instead of using a unique multi-objective function (the usual average weighted sum or product of the multiple objectives can become difficult, due to the interaction between the different objectives), the different objectives are decoupled in single objective functions, that are sequentially optimized in corresponding stages. All except the last stage are constraint optimization. Each solution is evaluated according to one single objective function, following a sequential order. The solution is evaluated using the objective function of a given stage until the constraint of that stage is fulfilled. Therefore, each evaluated solution is defined by a tuple *(s, v)*, where *s* is the stage reached and *v* is the fitness value obtained using the objective function of this stage. The solutions are then ranked according to their stages *s* and, within a stage, according to the value of the associated objective function *v*. In other words, assuming maximization, the following conditions hold:

- The stage are ordered so that a solution in a higher stage is always considered fitter.
- A solution can be in only one stage.
- Solutions in the same stage *s_j_* are ordered using the fitness function *f_j_* associated to that stage
- A solution is in stage *s_i_* with *i* > 0, if all the constraints associated to stage *j* < *i* are fulfilled but not the one of stage i.

Here we used 4 stages whose associated fitness functions and continuation criterion are given in Supplementary Table 4. The first stage optimizes for a walking gait that can cover at least a distance of *d*_lim_. Since the model can generate gaits of various speed, we add a second stage to constrain the speed of the walking solution so as to facilitate further comparison between different obtained solutions. The third stage minimizes a penalty term accounting for knee overextension to favor human-like gaits. The fourth stage minimizes the metabolic energy expenditure. The model used for calculating the energy expenditure is based on a model of the energy consumption of a muscle as described in Bhargava et al. (2004) and as used in Wang et al. (2012).

Since we want to add a feedforward component to modulate the gait, the initial model should have the capacity to manage changes in acceleration, deceleration or step lengths, i.e., should be robust. However, optimizing for energy consumption on a flat ground will not favor the emergence of such gaits. In order to circumvent this issue and favor robust solutions, we optimize the feedback parameters on an environment with increasing and decreasing slope. The increasing/decreasing slope are modeled as simple trapezoidal structure (with max slope 5%). Furthermore, the length, slope and distance between trapezoidal structure are randomized (details concerning the environment can be found in Dzeladini, 2013). During the optimization process, each solution is evaluated on 5 different randomly generated environments, and only the worst fitness score is considered.

### 2.4. FBL extension: 3FBL

The extended model is a hybrid feedback and feedforward model, referred to as 3FBL. The CPG component (IN_cpg_) generation is based on an idea from Kuo (2002), where feedforward signals produced by the CPGs are considered as feedback predictors. A direct way of combining such CPGs with feedbacks is to use a proportional term to control the relative importance of the CPG vs. the feedback it predicts, i.e., given the vector of CPG activities *X*_in_cpg__, Equation 2 representing the motoneurons states becomes:

(9) $$X_{\text{mn}}=G^{s}(\vec{\alpha }X_{{\text{in}}_{\text{sen}}}+(1-\vec{\alpha })X_{{\text{in}}_{\text{cpg}}})+X_{\text{mn}}^{0}$$

**Figure 2:**
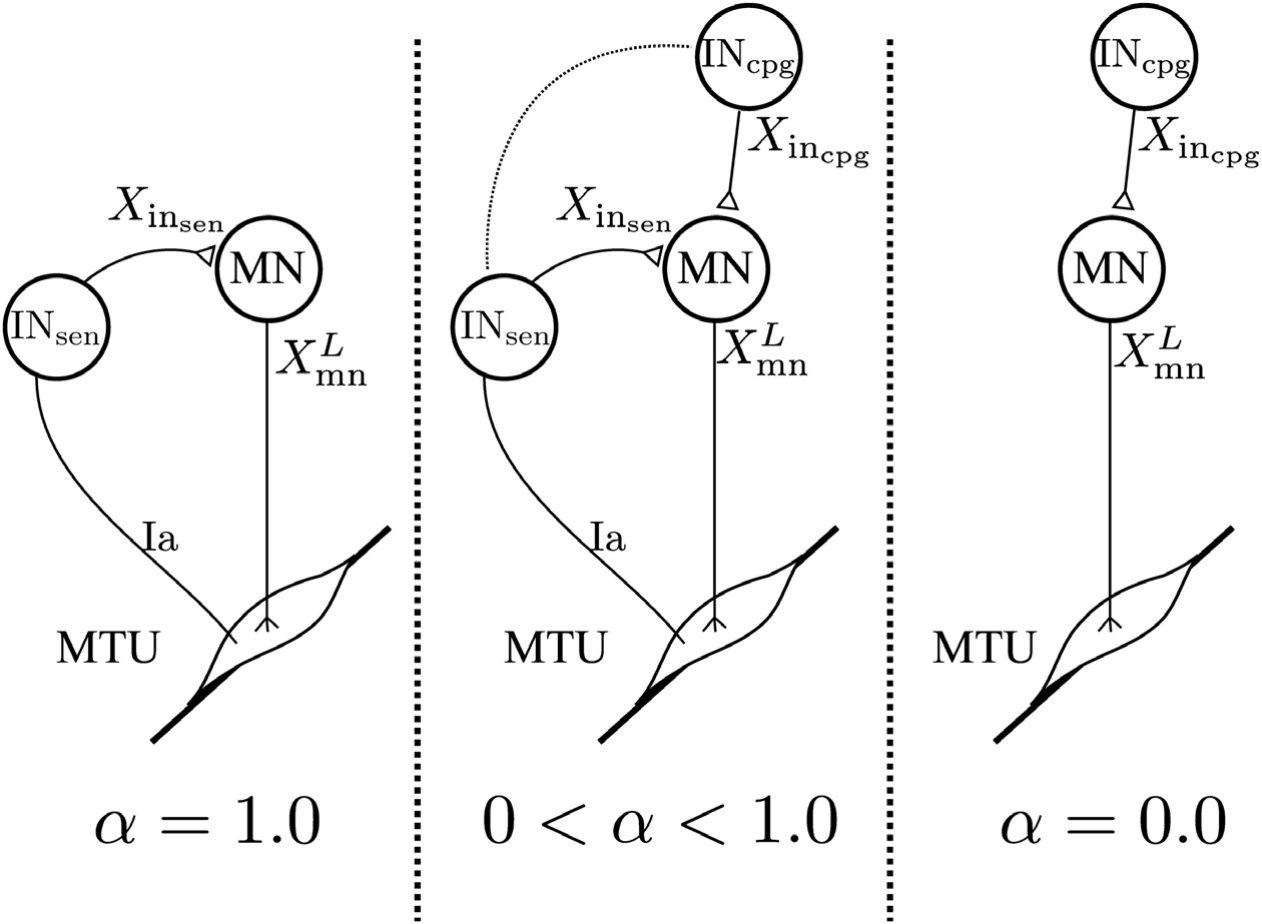
**Schematic representation of the spinal network for a specific feedback pathway**. The value of α controls the proportion of feedback vs. feedforward. With α = 1.0 the feedback pathway is solely feedback-driven. With α = 0.0 the feedback pathway becomes a feedforward pathway. All values in-between create a feedback/feedforward pathway.

Where: *G^s^*, *X*_mn_, *X*^0^_mn_ and *X*_in_sen__ are the same as in Equation 2. *X*_in_cpg__ is the vector of feedforward interneurons activities. Note that here *X*_in_cpg__ and *X*_in_sen__ have the same dimension but all the components of *X*_in_cpg__ referring to non-modeled sensory interneurons are set to 0. In the 3FBL models only the sensory interneurons related to muscles sensors are modeled with CPGs. Thereby, limiting the effective number of CPGs to 9 per limb. $\vec{\alpha }$ is a vector controlling the relative importance of sensory vs. CPG interneurons: a value of 0 in any of the α*_i_* components will make the corresponding pathway exclusively feedforward-driven, whereas a value of 1 would make it solely feedback-driven (see Figure 2). Thus, when $\vec{\alpha }$ = **1**, the 3FBL becomes the FBL model. Conversely, when $\vec{\alpha }$ = **0**, the activity of all the sensory interneurons is ignored and the model becomes a purely feedforward-driven model.

Any IN_cpg_ is by definition a model of the underlying feedback pathway IN_sen_. In this work we use two different abstract models of biological CPGs: a dynamical model IN^osc^_cpg_, generating periodic time varying signal and a constant model IN^cst^_cpg_, generating a constant signal (see section 2.5 for details). Both IN^osc^_cpg_ and IN^cst^_cpg_ can be viewed as a linear model of the underlying IN_sen_. The former is a model capturing the shape, timing and average activity while the latter only captures the average activity. Therefore, their combination with IN_sen_ can be viewed as a linearization of the underlying feedback pathways. Indeed, Equation 9 can be rewritten as:

(10) $$X_{\text{mn}}=G^{s}(X_{{\text{in}}_{\text{cpg}}}+\vec{\alpha }(X_{{\text{in}}_{\text{sen}}}-X_{{\text{in}}_{\text{cpg}}}))+X_{\text{mn}}^{0}$$

This representation highlights the fact that, in the 3FBL model, the equation governing the activity of the motoneurons can be viewed as a linear feedforward term, plus a corrective term (i.e., the difference between the IN_sen_ and IN_cpg_ state). As expected, the effect of a IN^osc^_cpg_-IN_sen_ combination is different from the one of a IN^cst^_cpg_-IN_sen_ combination. On the one hand, increasing the proportion of IN^cst^_cpg_ can be viewed as reducing the amplitude of the underlying IN_sen_, without affecting its mean activity. In other words, the proportion of IN^cst^_cpg_ vs. IN_sen_ controls the flatness of the IN_sen_. On the other hand, combination of IN_cpg_ and IN_sen_ will neither significantly affect the shape, nor the average activity of the IN_sen_, but will affect the timing.

### 2.5. 3FBL components

#### 2.5.1. CPG-Constant model

In order to test whether a very simple model of feedback could already capture enough information to permit modulation, we decided to implement a CPG-Constant model, denoted IN^cst^_cpg_. IN^cst^_cpg_ state, is a constant signal, whose value equals the average underlying IN_sen_ state. The average is calculated only on the part of the cycle where the feedback is active (e.g., for feedback active only during the stance, the average is calculated only during stance). This type of feedforward signal captures the average activity of the underlying feedback pathway. When combined with feedbacks (see section 2.4), the net effect is a flattening of the original feedback signal.

#### 2.5.2. CPG-oscillator model

In the oscillatory model, denoted IN^osc^_cpg_, each feedback predictor is modeled as a dynamical system reproducing the average shape and amplitude of the original feedback signal. In other words, CPGs can be viewed as a dynamical approximation of the sensory interneurons states *X*_in_sen__ (see Equation 1). The dynamical system used for this purpose is a morphed oscillator (MO) (Ajallooeian et al., 2013). This oscillator is able to produce any shape, as long as this shape can be represented by a function that is both 1-periodic and derivable. The differential equation governing the oscillator is the following:

(11) $$\dot{\theta }\;=\omega$$

(12) $$\dot{x}=\gamma \left(g(\theta )-x\right)+\frac{dg}{d\theta }\;\cdot \;\dot{\theta }+K$$

Where $\dot{\theta }$ is the frequency of the oscillator, γ (here set to 100) controls the speed of convergence of the oscillator output *x* toward the shaping function *g*(θ), and *g*(θ) is the nominal function that shapes the output of the oscillator, this function is extracted from IN_sen_ states, see next paragraph.

**2.5.2.1. *Pattern generation***. In order for the stability condition of the MO to be fulfilled, the pattern of the CPG must be represented by a first order differentiable 1-periodic function. Based on our hypothesis that CPGs can be viewed as feedback predictors, this function should reproduce the typical shape of the corresponding feedback pathway, for each cycle. The typical shape is derived as follow: (1) the sensory signals are recorded from a stable walking solution, (2) each sensory signal is split into cycles using the ipsilateral limb takeoff event (for feedback pathways active during swing), or the ipsilateral limb touchdown event (for all other feedback pathways), (3) each resulting sub signal is normalized in the temporal domain, in order to obtain a set of *N* repetitions of the sensory signal shape *p*(θ,*i*),*i* = [1,…,*N*], (4) the shaping function *g*(θ) is then derived using a third order spline interpolation of the mean signal.

(13) $$g[\theta ]=1/N\sum_{i=1}^{N}p[\theta ,i]$$

**2.5.2.2. *CPG coupling with the environment***. All oscillators have the same frequency ω initially set to an estimate of the FBL gait frequency from which the feedback patterns were extracted. In order to ensure that CPGs stay synchronized with the gait phases on which they should act, a coupling has to be defined. This coupling should ensure that:

1. IN_cpg_ will always start at the beginning of the gait phases during which it acts, at the touchdown/takeoff events of left limb for IN_cpg_ acting during left stance/left swing respectively, same holds for right limb. This event is called the synchronization event.
2. IN_cpg_ will never starts a new period before the gait phases on which it acts ends.

Consequently there should be four different oscillators driving the different IN_cpg_, i.e., two for each limbs: one that uses touchdown as synchronization event (used by IN_cpg_ acting during stance or whole cycle) and an other one that uses takeoff as synchronization event (used by IN_cpg_ acting during swing), Figure 2B shows the organization of the spinal network. Each oscillator is coupled to the environment using the following frequency adaptation mechanisms implementing the two requested coupling properties:

1. If the oscillator is too slow compared to the walking frequency, the phase of the central clock is simply restarted and set to 0.0 at the synchronization event (see Supplementary Figure 2A).
2. If the oscillator is going too fast compared to the walking frequency, a slowing down mechanism takes action before the expected synchronization event (see Supplementary Figure 2B). It ensures that signals generated by the MOs will not start a new cycle before they should (e.g., for oscillators active during stance, before the limb touches the ground).

With both mechanisms turned on, the phase of oscillator *i* is defined as:

(14) $${\dot{\theta }}_{i}\;=\{\begin{array}{ll} \omega & \text{ if }t_{i}<p\;\cdot \;\frac{1}{w}\\ c(t_{i}) & \text{ else } \end{array}$$

(15) $$\theta_{i}=0\text{ if }t_{i}>\frac{1}{\omega }$$

Where: θ_*i*_ is the phase of oscillator *i*, *t_i_* is the time since the last synchronization event and *p* is the percentage of the phase at which the slowing down mechanism is turned on. *c(t)* is a slowing down function that ensures that θ ⩽ 1.0, ∀ *t* ϵ ℝ For the slowing down mechanism to enter in action after 90% of the period of the oscillator (i.e., *p* = 0.9), we can use the following function:

$$c(t_{i})=10\omega \;\cdot \;exp\;(-10\omega t_{i}-ln(10)+9)$$

Details on how *c(t)* is derived can be found in Dzeladini (2013).

#### 2.5.3. Feedback sensitivity scale

For a feedback pathway *i*, the feedback sensitivity is noted *FDB^sen^_i_* = 1 − α*_i_* and corresponds to the point at which the gait becomes unstable when (1) all other feedback pathways are kept as feedbacks (i.e., α_*j*_ = 1 for all *j*≠ *i*) and (2) the feedback pathway *i* is combined with an IN^osc^_cpg_. A feedback sensitivity of 0 means that the feedback can be fully replaced by its cognate IN^osc^_cpg_ predictor without destabilizing the stability of the generated gait.

### 2.6. 3FBL models

In order to demonstrate the effect of feedback and CPG combinations, we created different models combining CPG and feedback components in different ways. Here we present only the 5 models exhibiting the most interesting properties in terms of speed modulation. The 5 models differ in their CPG-feedback combination vectors $\vec{\alpha }$ (see Table 3 for details). Contrary to what might be expected, a 3FBL model with a IN^osc^_cpg_-IN_sen_ combination vectors of 0.5 for all muscle feedbacks pathways was not good in terms of speed modulation when considering global control variable acting on all CPGs. The first 4 models study the effect of a CPG addition on different group of muscles, namely the 3FBL^osc^_ankle_, 3FBL^osc^_hipA_, 3FBL^osc^_hipB_, and 3FBL^osc^_biArt_. The fifth model, referred to as 3FBL^min^_fdb_, is a minimum feedback gait, designed to study the properties of gait with minimal feedback activity. That model was obtained as follows: IN_cpg_ are added starting from pathways acting on distal muscles. Pathways acting on distal muscles use CPG-CST models (IN^cst^_cpg_) and pathways acting on proximal muscles use CPG-OSC models (IN_cpg_), using the lowest possible α (in the [0, 1] range). This methodology was chosen, with the aim of finding a gait with the minimal number of feedbacks. Note that other CPG-FDB combinations might be found using different methodologies. Using this methodology, the 3FBL^min^_fdb_ gait generated stable walking, with a feedback activity corresponding to 35% of the IN_sen_ related to muscle feedbacks, and 45% of all the feedbacks (the feedback activity is defined as $\frac{\sum_{i}\left(\alpha_{i}\right)}{N}$, where *N* is the number of feedbacks).

**Table 3:**
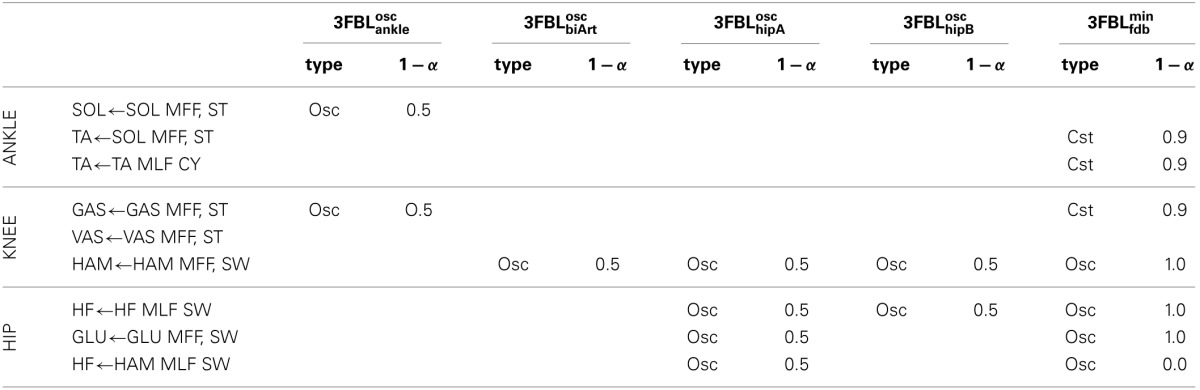
**Description of the CPG-FDB combination map for the 5 different 3FBL models**.

*Each row shows for a given feedback pathway, the type of CPG used (Osc stands for IN^osc^_cpg_ and Cst for IN^cst^_cpg_) and the level of CPG-FDB (i.e., α) for the 5 different 3FBL models. The four first columns shows the most interesting 3FBL models in terms of speed control: 3FBL^osc^_ankle_, with CPGs acting on distal extensor muscles, 3FBL^osc^_hipA_ and 3FBL^osc^_hipB_, with CPGs acting on HIP muscles and 3FBL^osc^_biart_, with CPGs acting on the HAM bi-articular muscles. The last column shows the combination vector for 3FBL^min^_fdb_ (i.e., the minimum feedback model). Note that the 3FBL^min^_fdb_ also replaces the “VAS←GCF STend” and “HF←TLF SW” pathways by a CPG-CST predictor. Note that only the pathways related to muscle feedbacks are shown. Even though a full replacement of the “VAS←GCF STend” pathway by CPG-CST is possible without affecting the produced gait, the effect of a modulation produces no significant effect on the resulting gait (data not shown). This pathway is thus not used, except for the 3FBL^min^_fdb_. The KNEE overextension prevention pathway (“VAS←KNEE OPF”) and the pathways related to stability (i.e., “HF←GSIF ST,” “HAM←GSIF ST”, “GLU←GSIF ST” and “HF←TLF SW”) are not used, as their role as feedback is evident. Moreover, even though a combination with CPG generates stable walking, walking becomes unstable even with very small modulation of the CPG parameters (data not shown)*.

### 2.7. 3FBL modulation : model of supraspinal influences

**Figure 3:**
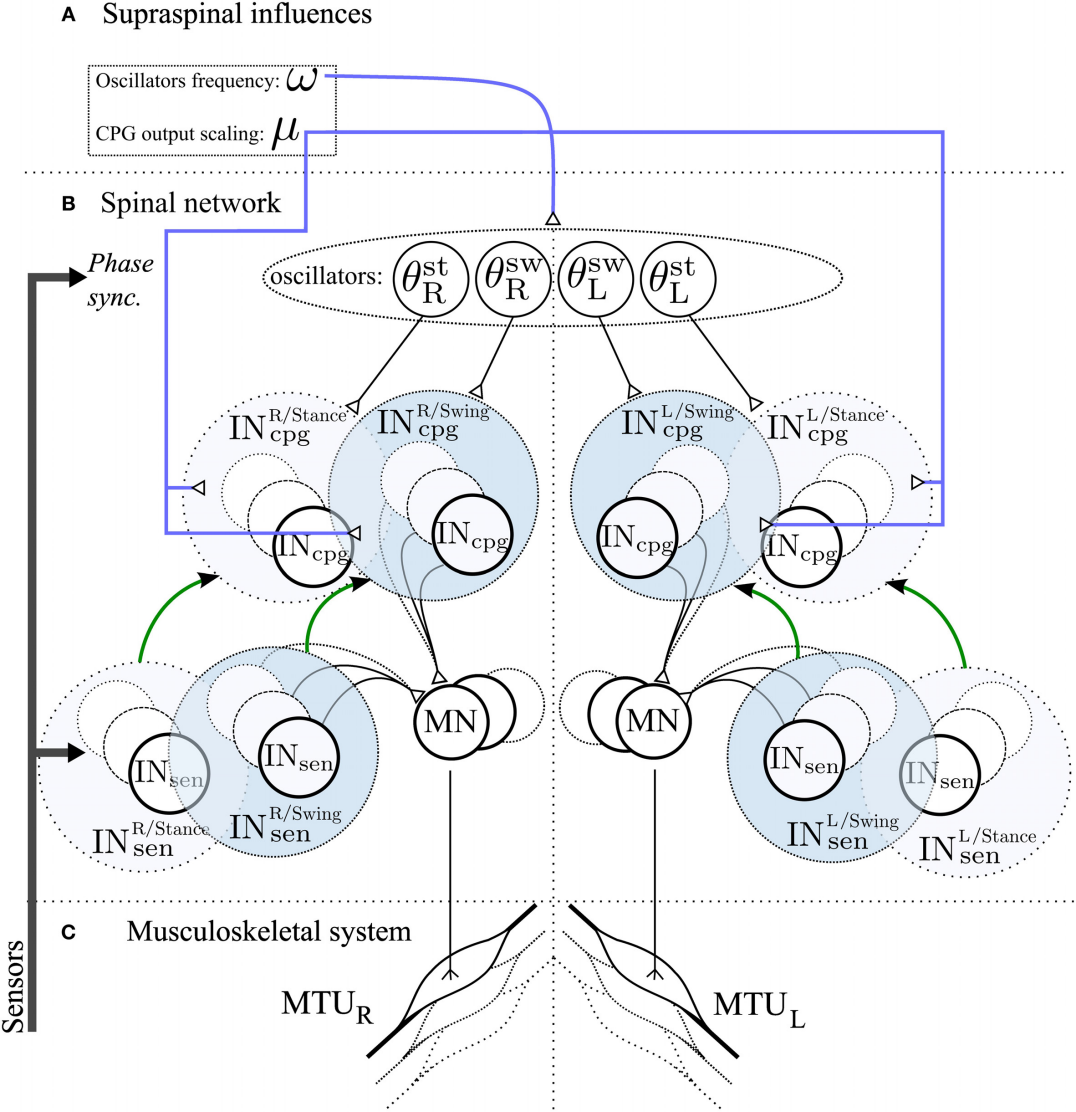
**Schematic representation of the spinal network and supraspinal control of the CPG network in the 3FBL model**. The network is symmetric: left/right part of the figure corresponds to the part of the network acting on right/left limb muscles respectively. **(A)** Suprasinal influences: μ represents the activity modulation pathway and ω the frequency of the CPG network. All 4 oscillators share the same ω, but each CPG can have a different μ. If not stated otherwise, all IN^osc^_cpg_ and IN^cst^_cpg_ share the same amplitude modulation μ_*osc*_ and μ_*cst*_, respectively. **(B)** Spinal network. Four oscillators, differing in their synchronization mechanism with the environment, drive the different IN_cpg_. θ^st^_R_,θ^sw^_R_,θ^sw^_L_ and θ^st^_L_ are used by IN_cpg_ starting at right limb stance, right limb swing, left limb stance and left limb swing respectively. IN_cpg_ and IN_sen_ action on MN follows Equation 10. The green arrow between Sensory and CPG Interneurons pathway highlights the fact that each CPG pathway is a model of one sensory pathway. **(C)** Musculoskeletal system, there is one muscle corresponding to each individual motoneurons.

We hypothesize that the use of a CPG component will facilitate speed control. Indeed, it is known that simple supraspinal signals are sufficient to modulate gait frequency in lower vertebrates and in mammals, as demonstrated by experiments on decerebrated cat walking on a treadmill, where speed changes and gait transitions can be elicited by varying the stimulation of the mesencephalic locomotor region. We model two different kinds of descending pathways (see Figure 3):

- Frequency : ω
  Controls the frequency of the CPG-OSC (ω value in Equation 14). This variable affects all oscillators as they share the same frequency.
- Activity modulation : μ
  Modulates the CPG activity of both CPG-OSC and CPG-CST. Effectively, the CPG output *X*_in_cpg__ becomes μ · X_in_cpg__, with μ > 0 controlling the activity of the CPG.

## 3. Results

The results are separated in three parts. In the first part, we compare the gait produced by the optimized FBL model with human walking, in terms of metabolic cost, gait harmony and gait kinematics. In the second part, we present an analysis of the different feedback pathways of one specific solution of the FBL model. We analyse each feedback pathway separately and for each of them study the effect of a combination with their feedforward predictor. Finally, in the last part, we analyze the 3FBL models in terms of speed control.

### 3.1. FBL: feedbacks based locomotion model

In order to determine the ability of our optimization process to generate stable gait, we performed 10 runs of the same optimization process (as described in section 2.3) with different random initial condition. We observe that the optimization process always converges to a stable and symmetric walking solution, but to different solutions (local optima), hence leading to visually different gaits. **Figure 5F** gives a snapshot of the solution 1 during two cycles. Note that the presented results are, in terms of joint angles, joint torques and muscles activities, qualitatively similar to those presented in the paper describing the original model (Geyer and Herr, 2010).

#### 3.1.1. Metabolic cost analysis

**Figure 4:**
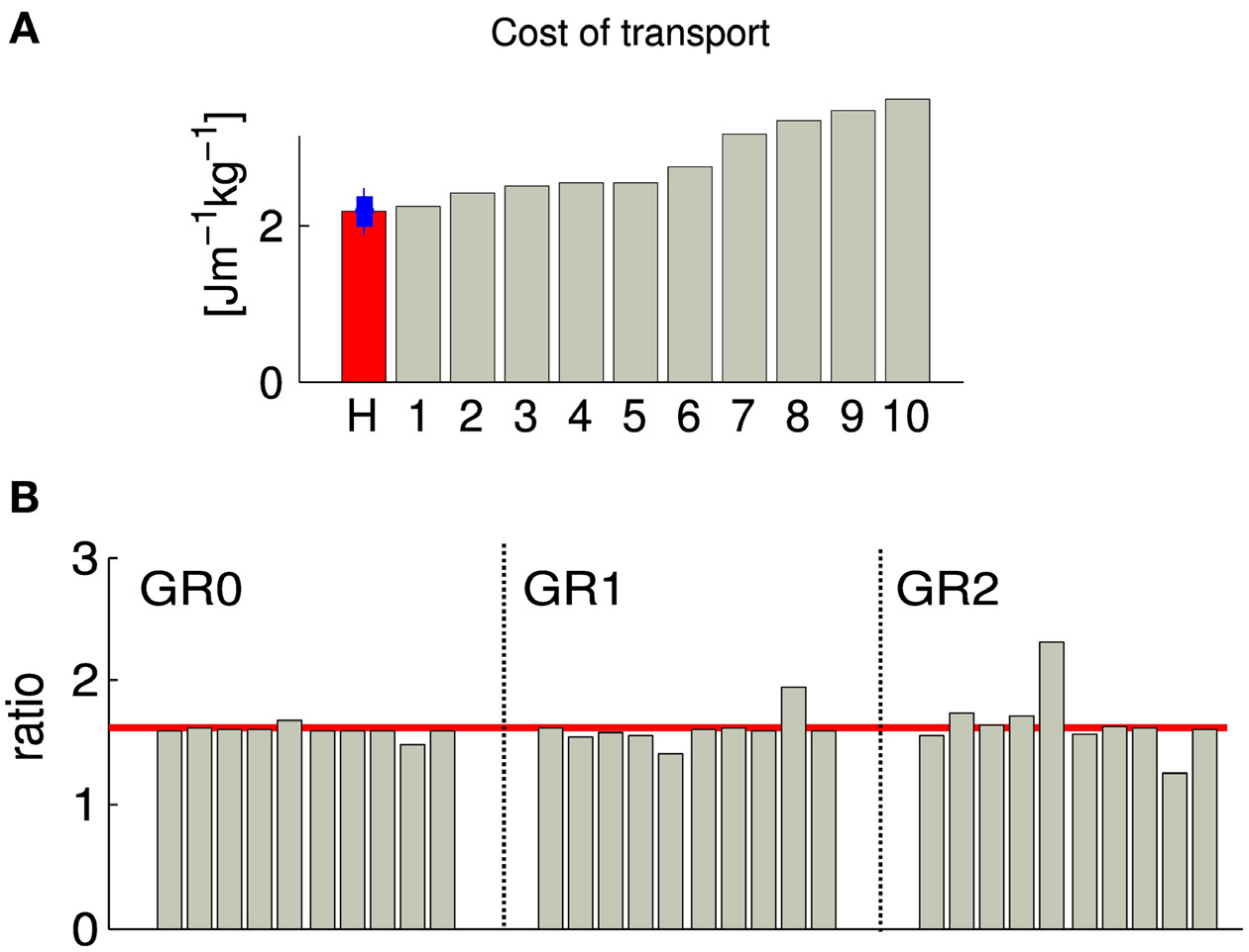
**Each gray bar corresponds to one solution of the same optimization process (optimizing for a stable gait walking at 1.3 m/s)**. **(A)** Normalized cost of transport. The red bar corresponds to the normalized cost of transport of human subject of the similar weight and walking speed as our obtained gait (data from Weyand et al., 2010), the blue bar shows the estimated standard deviation. **(B)** Duration proportion of the different gait phases. GR0 corresponds to the ratio between cycle duration and stance duration, GR1 corresponds to the ratio between stance duration and swing duration and GR2 corresponds to the ratio between swing duration and double stance support. The red line corresponds to the golden ratio $\phi =\frac{1+\sqrt{5}}{2}$. GR0, GR1, and GR2 are known to be statistically similar to the golden ratio in human walking at their preferred speed (Iosa et al., 2013).

When comparing the cost of transport (CoT) between the 10 different solutions, we observed a value ranking from 2.2 to 3.5 [Jm^−1^kg^−1^] (CoT is defined as *E/md*, where *E* is the energy consumed during the run, *m* is the mass of the model, *d* is the traveled distance), see Figure 4. Five solutions show a CoT less than 25% higher than the net metabolic transport cost of ~2.1 [Jm^−1^kg^−1^] found in human subjects of similar heights, weights and walking at the same speed (Weyand et al., 2010). This increase is comparable to the one found in Bhargava et al. (2004) and can be explained by the fact that, in our model, the upper body is modeled as a single rigid body, while the experimental values used for comparaison are for walking with arm swing. Indeed, it has been shown that, despite the fact that arm muscles consume energy to produce movement, they can still reduce the walking metabolic cost up to 12% (Collins et al., 2009). An other reason explaining the higher CoT could be the lack of feedbacks for stance preparation. Indeed, as most of the metabolic cost of walking comes from the stance phase, optimizing the properties of the limb joints before touchdown will affect the efficiency of walking, as shown in Donelan et al. (2002).

#### 3.1.2. Golden ratio analysis of gait harmony

As demonstrated in Iosa et al. (2013), the ratios between cycle/stance durations (noted GR0, commonly referred to as the duty factor), stance/swing durations (noted GR1), and swing/“double stance support” durations (noted GR2) is similar in healthy humans of different size, corpulence and age walking at preferred (self-chosen) speed, and satisfy the golden ratio ($\sigma =\frac{1+\sqrt{5}}{2}$). Note that the variability of GR1 is higher than GR0, and the variability of the GR2 is higher than GR1. We measured those three ratios in our 10 solutions, and observed that GR0 converges to σ in all cases, GR1 converges to values close to σ with higher variability and a bias to slightly smaller values, and GR2 is more variable, with a bias to values higher than σ. The bias observed in the cases of GR1 and GR2 indicates that there is a tendency to generate gaits with longer swing and shorter double stance support phases. This overestimation of the swing duration can be explained by the fact that our model does not have toes; the length of the foot being shorter, the legs tend to enter the swing phase earlier.

#### 3.1.3. Gait analysis

**Figure 5:**
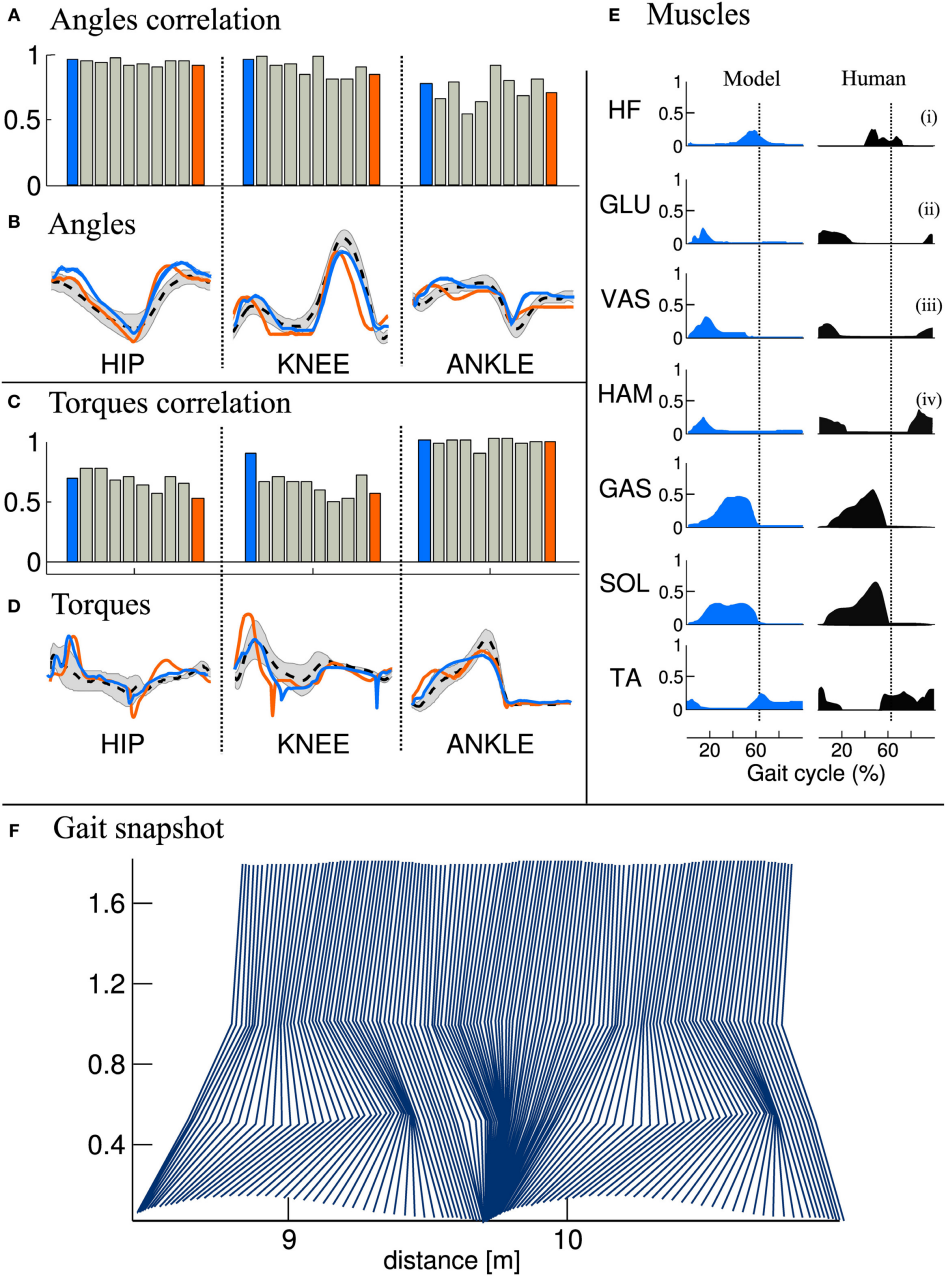
**Comparison of joints angle, joints torque and muscles activity extracted from the FBL models (10 optimization runs), with human data**. Human joints angle and torque are taken from Winter (2009), muscles activities are adapted from Perry et al. (1992), as presented in Geyer and Herr (2010). **(A)** Joint angle correlation with human, **(B)** Average joint angle compared to human, **(C)** Joint torque correlation with human, **(D)** Average joint torque compared to human, **(E)** average muscles activity of solution 1 compared to human and **(F)** Gait snapshot of the solution 1 over two cycles. In **(A,C)**, the bar plots show the correlation with human for the different solutions and for the different joints. In **(B,D)** are shown typical human trajectories (black dotted line:mean, gray: standard deviation) and two mean trajectories from solution 1 and solution 10, blue and orange lines respectively. Each bar corresponds to one solution of the same optimization process (optimized for a stable gait walking at 1.3 m/s), the different solutions are ordered with increasing energy consumption (same as in Figure 4). The correlation were calculated on data extracted from 50 strides of steady state walking (sampling frequency of 1 Khz), spline interpolation was used to normalize the length of the vectors to 1000 points. The average of the normalized vector was then correlated with average human data. In **(E)** the subscripts show the compared muscles: (i) adductor longus, (ii) upper gluteus maximum, (iii) vastus lateralis, and (iv) semimembranosis. Note that the data was extracted from a model walking on a flat terrain without noise and external perturbations. Therefore, the standard deviation of the angles and torques trajectories and muscles activities is very small and thus not visible.

We then compared the joint angles and torques trajectories of the 10 solutions, with human data (Winter, 2009). A correlation analysis revealed that all joints angles and torques are comparable to human data (see Figures 5A,C, if not stated otherwise, the solutions are ordered with increasing CoT). While the ANKLE torques show high correlation with humans, the HIP and KNEE torques correlations are substantially lower. This can be explained by the fact that, in our model, the HIP is completely fixed to the trunk. We thus do not model the characteristic pelvis movement observed in human walking. Regarding the joint angle correlations, we can see that the ANKLE angle correlation is not perfect. The low correlation can be explained by the differences in shape in late stance and early swing (see Figure 5B, right), which is due to the fact that the toe is not modeled. Indeed, the lack of toes will make the leg enters in swing earlier, thereby explaining both the reduced minimum angle and the earlier slope inversion (i.e., the swing/stance transition).

Another interesting difference between the model and human data can be noted at the ANKLE angle level during early stance. Indeed, while humans show an initial passive extension during early stance of about 1/10th of stance duration (black dotted line in Figure 5B right), the model does not show this behavior. When looking carefully at the ANKLE angle pattern for solution 1 an initial passive extension is visible. However, this initial passive extension is very short and almost not visible in the figure (blue line in Figure 5B right, the ANKLE angle does not start at the same place due to a very fast and quick passive extension). The solution 10 (orange line in Figure 5B) does not show this behavior at all: the foot touches the ground horizontally. Several elements can explain this behavior, such as the lack of mechanism (e.g., feedback, CPG) for stance preparation, a shorter swing range (due to smaller HIP range or an under-extension of the knee) or the way the swing-stance transitions are designed, i.e., state machine with discrete transition.

When comparing muscles activities of solution 1 (see Figure 5E), we note that all the ANKLE muscles and HF muscle are close to human data. However, the GLU, VAS and HAM muscles do not show the typical activity observed during late swing in humans. This is in agreement with the conclusion drawn in the previous paragraph concerning the lack of a mechanism for stance preparation.

### 3.2. Feedback pathways study

#### 3.2.1. In_sen_ signal analysis and prediction

Since the produced gaits are all symmetric and stable (i.e., close to perfectly periodic), the feedback signals should be very similar between cycles. Consequently, the quality of the feedback prediction should be very high (i.e., IN^osc^_cpg_ should be very close to IN_sen_). In order to study the quality of the prediction, we generated the IN^osc^_cpg_ (as described in section 2.4) and ran them in a passive mode (no action on muscles, i.e., no link between IN^osc^_cpg_ and MN). The Supplementary Figure 1 shows the actual IN_sen_ signals (dotted lines) and the reproduced signal (thick lines) over one step, for the worst gait (in terms of feedback prediction quality, i.e., similarity between IN_sen_ and IN^osc^_cpg_). We can see that the prediction is very close to the feedback signals; the lowest correlation between the original and the reproduced signals is of 0.98. Differences are noted as shifts and amplitude differences, and are due to small asymmetries in the gait. It is interesting to note that, even if those asymmetries are visible at the level of the feedbacks, their effects on the gait are very small. However, even small asymmetries between the IN_sen_ and their predictors (IN^osc^_cpg_) can create instabilities which makes their replacement difficult.

#### 3.2.2. Feedbacks replacement

In order to study the possibility of replacing the feedbacks (IN_sen_) by their full predictors (IN^osc^_cpg_), we ran a systematic search in which we increase β = 1 − α (i.e., the proportion of IN^osc^_cpg_) from 0 to 1.0 using the combination strategy presented in section 2.3.3. The systematic search is done for each feedback pathway *i*, where β*_i_* is increased from 0 to 1 in steps of 0.1. All the others pathways are kept as feedbacks (i.e., β_*j*_ = 0, *j* ≠ *i*).

The Supplementary Table 2 shows, for each gait, the number of feedback pathways that could not be fully replaced (i.e., the feedback pathways that have a *FDB^sen^_i_* ≠ 0. Table 4 shows the feedback sensitivity of the 7 best gaits, in terms of the number of feedback pathways that can be replaced, i.e., in terms of feedback replacement capacity (see section 2.5.3 for details on the feedback sensitivity scale). It is interesting to note that feedback pathways acting on ANKLE muscles have a zero feedback sensitivity value which means that they can be fully replaced by a IN^osc^_cpg_ model without loss of stability. The muscle length feedback pathway from HAM bi-articular muscle acting on the HF muscle always shows a high sensitivity (for gaits showing meaningful CoT), highlighting its importance for the stability of the gait. Even though feedback related to trunk stability (feedback type 4) are crucial to ensure stable walking and to enhance gait resistance to perturbations, they are not part of sensitive feedbacks. However, a gait with only one trunk stability feedback replaced is stable only in steady state walking; as soon as small perturbations (pushes and/or change in slope) are exerted on the model, the gait becomes unstable and falls.

**Table 4:**
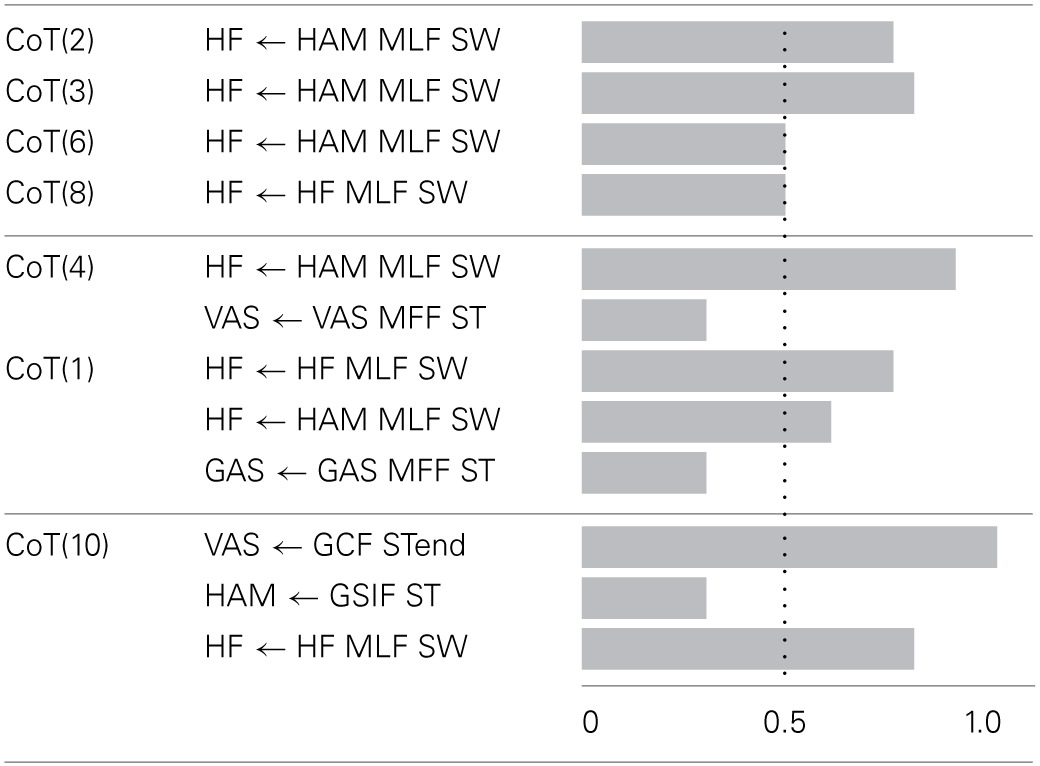
**Feedback sensitivity (see section 2.5.3) for the best 7 solutions (in terms of IN_sen_ replacement capacity, i.e., percentage of IN_sen_ that can not be replaced by a CPG-OSC model)**.

*The first column shows the solution, ranked in term of cost of transport (CoT). The second column gives the name of the feedback pathway. The third column shows the feedback sensitivity (FDB^sen^)*.

Based on these results, we focus on gait 2, as it shows a good correlation with human data, a meaningful CoT and a low feedback sensitivity, for further analysis.

#### 3.2.3. Feedbacks combination

**Figure 6:**
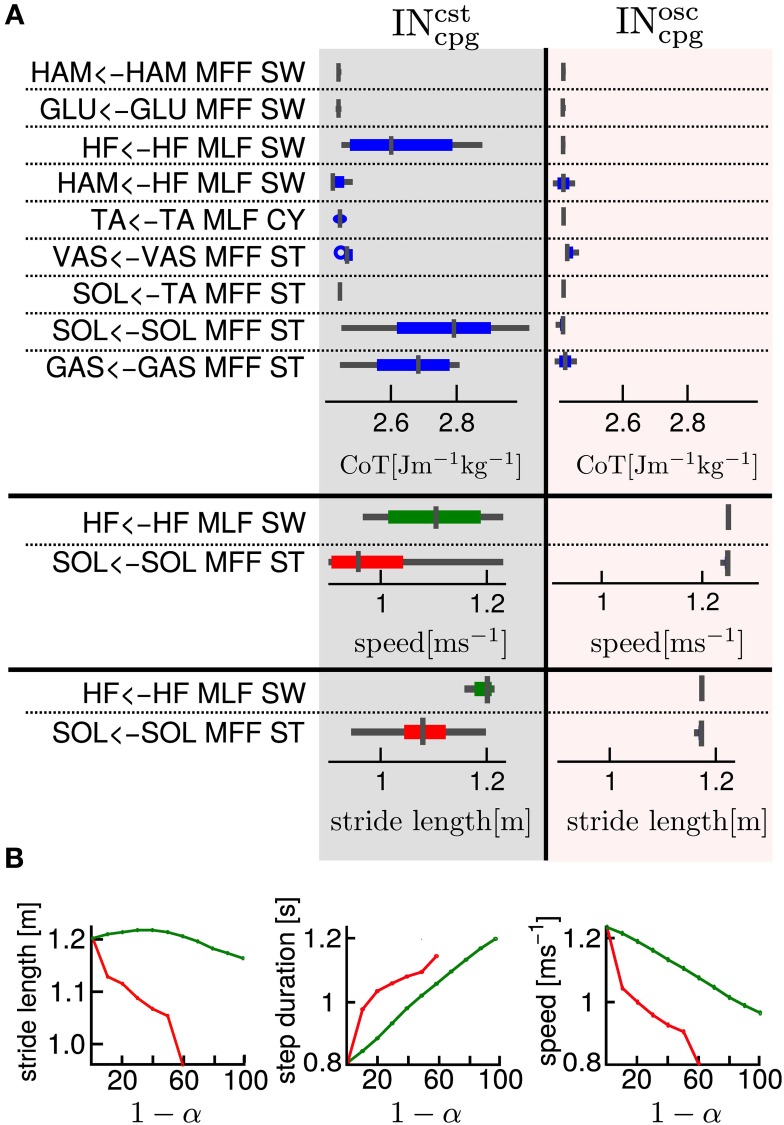
**(A)** One by one feedback and feedforward combination effects on cost of transport, stride length and speed, for gait number 1. The first column gives the name of the feedback pathway considered. The second and third columns show for an IN_sen_-IN^cst^_cpg_ and an IN_sen_-IN^osc^_cpg_ respectively, a box plot of the variation of a measured variable when α varies from 1 to 0. In the first part of the table the considered variable is the cost of transport (CoT), in the second part, the speed and in the third part, the stride length. We show the speed and stride length box plot only for the two most interesting pathways in terms of feedback and feedforward combination effect on CoT. The box plot read as follow: the middle line is the median, the colored line represents 99% of the data assuming the data are normally distributed and the gray horizontal bar shows the range of the measured variable. A very thin box plot (no colored line visible) means that the variation of α had no effect on the considered variable, feedback pathway and IN_cpg_ model. As expected the IN_sen_-IN_cpg_ combination for any α in the [0, 1] interval has very little effect on the CoT. **(B)** Relationship between IN^cst^_cpg_ proportion and gait variables, for two selected feedbacks (red, “SOL←TA MFF, ST” and green, “GAS←GAS MFF, ST”). Left: relationship between stride length and 1 − α (i.e., the IN_cpg_ proportion), Middle: relationship between step duration and 1 − α and Right: relationship between speed and 1 − α.

Figure 6A shows the effect on the generated gait (in terms of CoT, stride length and speed) of an increase in the proportion of feedforward vs. feedback signal for one specific pathway while maintaining all the other pathways purely feedback driven (this was implemented by decreasing the feedback proportion by steps of 0.1 of one component of the $\vec{\alpha }$ vector at a time while keeping all other components at 1). Figure 6A Left and Right parts show the combination analysis of feedbacks with IN^cst^_cpg_ and IN^osc^_cpg_ respectively. As expected, the replacement of IN_sen_ by a constant model (i.e., IN^cst^_cpg_) has more effect on the gait characteristics, compared to the replacement of the IN_sen_ by an oscillatory model (i.e., IN^osc^_cpg_). This confirms that the latter captures more information from the IN_sen_ (i.e., the shape, timing and amplitude).

Despite the higher sensitivity of the IN^cst^_cpg_-IN_sen_ combination (i.e., percentage of IN_sen_ that could not be replaced by a constant model (IN^cst^_cpg_), several interesting effects of the IN^cst^_cpg_-IN_sen_ combination are noted, as shown in Figure 6. We observe that, for the “SOL←TA MFF, ST” and the “HF←HF MLF, SW” feedbacks, changes in α (i.e., proportion of IN^cst^_cpg_ vs. IN_sen_) produce large variations in speed and stride length. In the case of “SOL←TA MFF, ST”, there is a linear relationship between the IN^cst^_cpg_ proportion level and both the speed and the stride length. A decrease in stride length and speed is observed with the increase in IN^cst^_cpg_ level, see Figure 6B.

### 3.3. 3FBL models : feedforward and feedback based locomotion model

In the previous section, we showed that all feedbacks can be combined with their CPG predictors, and that interesting properties, such as speed and step length variation, can be achieved, by playing with the CPG-FDB combination level when using CPG-CST predictors. While, in the previous section, feedback and CPG combinations were studied one pathway at the time, here we study effect of more complex combinations on 5 different 3FBL models exhibiting the most interesting property in terms of gait speed modulation (see section 2.6 for details).

#### 3.3.1. 3FBL^min^_fdb_: minimal feedbacks gait

**Figure 7:**
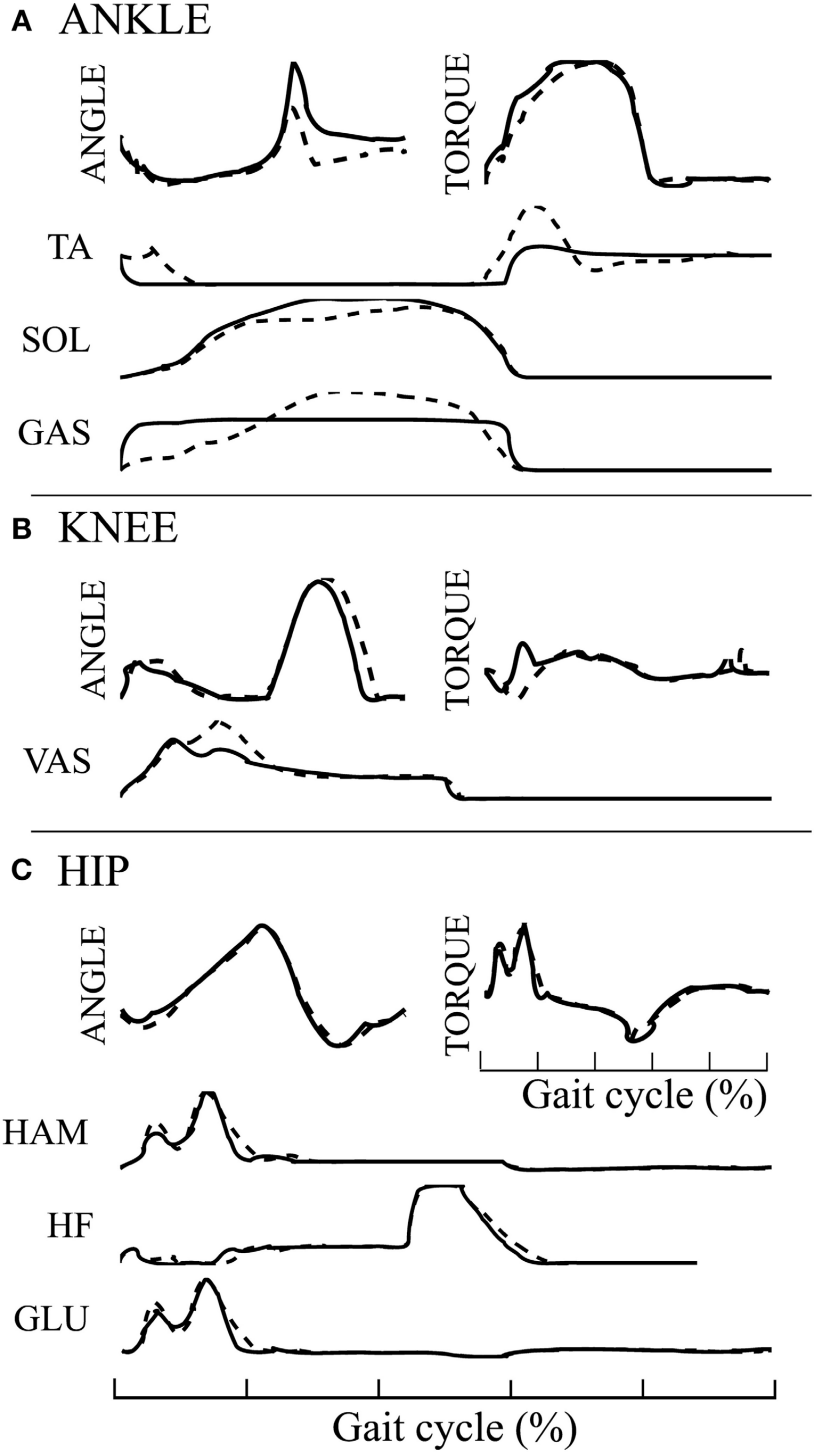
**Comparison of average joint angles, joint torques and muscles activation pattern between the 3FBL^min^_fdb_ (black line) and the FBL models (dashed line) for solution 1**. **(A)** ANKLE angle, torque and associated muscles activation level, **(B)** KNEE angle, torque and associated muscles activation level, and **(C)** HIP angle, torque and associated muscles activation level.

The 3FBL^min^_fdb_ model is able to produce stable walking with a global feedback activity reduced from 100 to 45%. Its average speed on flat ground is 1.35[m/s] (3% increase compared to the underlying FBL model). When comparing the joint angles, torques and muscles activities between the two models, almost no differences can be observed at the HIP joint (see Figure 7C). However, differences are noted at the level of the ANKLE joint (see Figure 7A). Indeed, all muscles activities acting on the ANKLE joints show different muscle activation patterns than the corresponding FBL model. Interestingly, the differences observed in muscles activities do not produce important changes in the shape of the torque and angle patterns of the ANKLE joint. Nevertheless, the increase in extensor muscles activities produces a steeper increase in joint torque during stance. This increase in torques explains the observed increased ANKLE angle at takeoff. In turn, this increase in ANKLE angle also increases the duration of the stance phase, thereby explaining the observed shift of the KNEE pick angle in early swing (see Figure 7B).

The SOL muscle shows a different muscle activation pattern, while the “SOL←SOL MFF, ST” pathway, the only one acting on it, has not been replaced by a CPG (i.e., kept as pure feedback, α = 1). Since the 3FBL^min^_fdb_’s feedback / CPG combination map does not permit a combination of CPG-OSC with feedback for this specific pathway (even with α = 0.95, i.e., pathway kept almost purely feedback), this change in activity is necessary to ensure a stable walking gait. This highlights the important stabilizing role that muscle feedbacks play in locomotion.

It is important to note that, while in a stable walking regime reducing as much as possible the proportion of feedback signals for specific pathways does not significantly affect the generated gait, the replacement of feedbacks considerably reduce the gait robustness to perturbations. Indeed, recovery after 0.25[s] pushes is reduced from 40[N] to 28[N] compared to the original gait. This highlights the importance of feedback to adapt to perturbations.

Even though the 3FBL^min^_fdb_ is valuable, as it shows that a large part of feedbacks can be removed from the FBL model, while a stable walking gait is still produced, it is not surprising that its modulation is almost impossible. Indeed, since a large part of the feedbacks are removed, even small modulations of CPG parameters render the gait unstable.

#### 3.3.2. Systematic study of supraspinal signal modulation and their effects on gait

Using the model of supraspinal influences presented in section 2.7, we ran a systematic search on the effect of CPG amplitude and frequency modulation on the different 3FBL models presented in the previous section, using ω and μ_osc_ as parameters (the parameters are split into 11 values across a given range ([0.2, 2.5] for ω and [0.1, 4.0] for μ_*osc*_).

**Figure 8:**
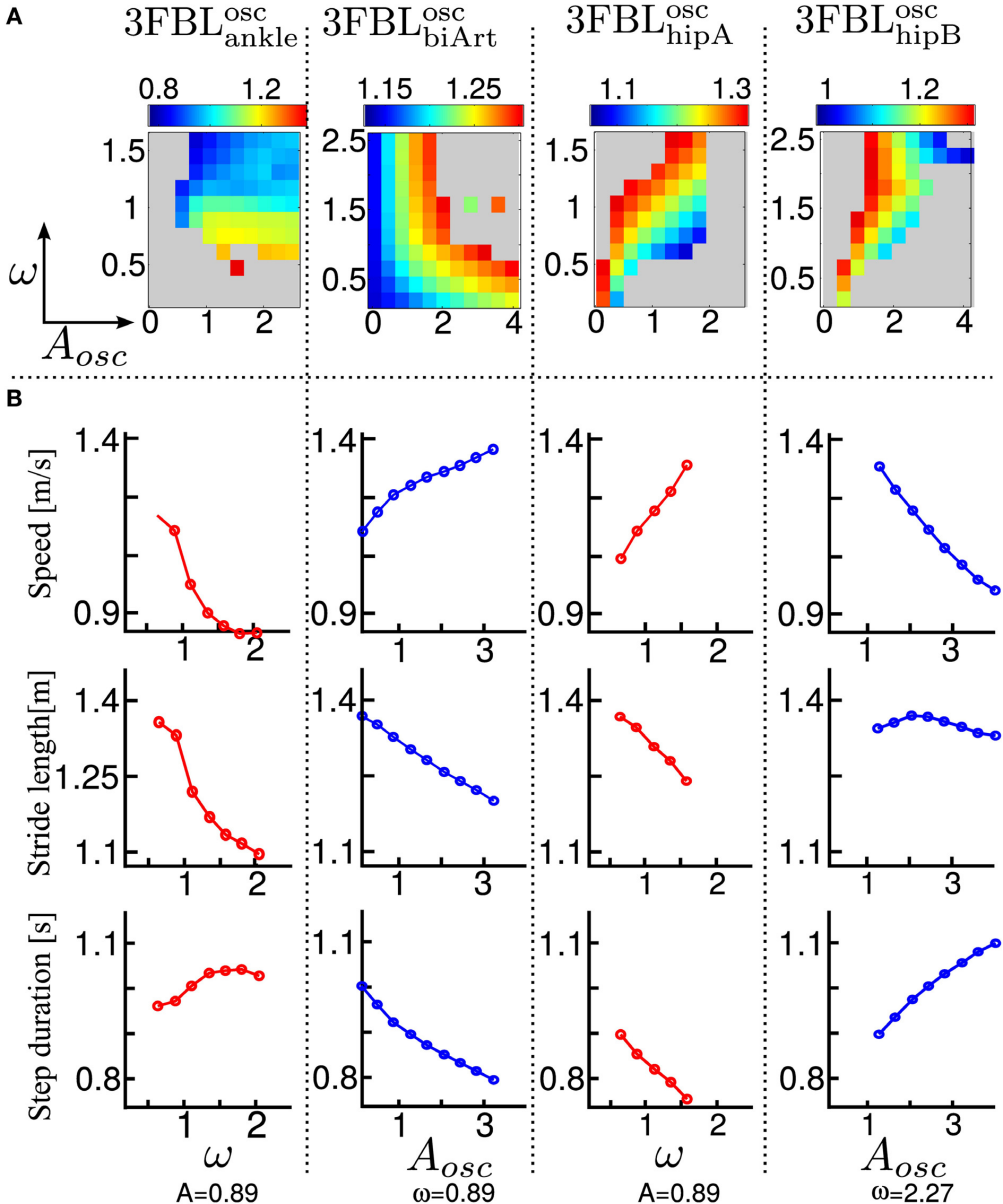
**Systematic search study of CPG parameters (supraspinal influences) for the different 3FBL models**. The systematic search is done for two parameters: ω, the frequency of the CPG network and μ_*osc*_, the CPG-OSC amplitude modulation. Each column corresponds to a given 3FBL model (name at the top, see Table 3). **(A)** Heat map of the systematic search. The color indicates the speed of the gait for a given (μ_osc_,ω) pair (gray color means that the gaits was unstable or asymmetric). **(B)** Highest variation in speed possible while maintaining one of the parameters constant (based on the heat map). A red/blue line means that (μ_osc_/ω) is kept constant, respectively. The value of the constant parameter is indicated at the bottom. The first row shows the speed, the second the stride length, and the third the step duration. Note that the 3FBL^min^_fdb_ is not shown as its modulation is almost not possible.

The systematic search on the 4 chosen models acting on different group of muscles (see Figure 8A) indicates that all the models are stable in a large range of amplitudes and frequencies, except the 3FBL^osc^_hipA_, that shows a more restricted region of stability. This can be due to the fact that the 3FBL^osc^_hipA_ has more oscillators than the three other models. Note that the restricted region of stability does not imply a restricted range of speed. Indeed, small variations in ω (while μ_*osc*_ remains fixed) induces noticeable change in speed in this model; an increase in speed is observed with an increase in frequency. In other words, changing the frequency of the 3FBL^osc^_hipA_ is sufficient to entrain the whole musculoskeletal system. Interestingly, this model—which is the only model with a high number of CPGs acting on proximal muscles—is the only one that shows an increase in speed when increasing the CPG network frequency. This suggest that CPGs acting on proximal muscles are required to produce a frequency-driven entrainment of the system.

Interestingly, the 3FBL^osc^_hipB_—which has only two CPGs acting on proximal muscles, compared to four in the case of the 3FBL^osc^_hipA_—shows almost no change in speed when the frequency ω is modulated (while μ_*osc*_ is fixed). Possibly, the frequency modulation of only two CPGs at the HIP level is not sufficient to produce a frequency-driven entrainment of the system. However, increasing μ_*osc*_ leads to a significant decrease in gait velocity. This decrease in speed with increasing amplitude is likely an effect of the “HF←HF MLF, SW,” as this effect is not observed in the 3FBL^osc^_biArt_, which differs from the 3FBL^osc^_hipB_ model only by the absence of a CPG component for this feedback pathway. Indeed, the “HF←HF MLF, SW” is a negative feedback, and thus increasing the amplitude of its associated CPG (i.e., μ_*osc*_) will reduce the activity of the HF muscle, reducing the HIP flexion velocity and hence increasing the duration of the swing, which in turn decreases the gait speed (as the stride length does not change significantly).

Surprisingly, as little as one oscillator is sufficient to allow significant changes in speed (shown by the 3FBL^osc^_biArt_, see Figure 8B). The changes in speed are mainly induced by a modulation of the amplitude μ_*osc*_, but with an opposite effect compared to the 3FBL^osc^_hipB_ (i.e., an increase in μ_*osc*_ leads to an increase in the gait velocity). However, since this effect is accompanied with a shortening of the stride length, this model is unlikely to be relevant; indeed, in humans an increase in speed is usually concomitant to an increase in stride length (Murray et al., 1966).

Note that small changes in speed are still possible with a modulation of the frequency ω, both in the case of the 3FBL^osc^_biArt_ and 3FBL^osc^_hipB_, but to a lesser extent than the 3FBL^osc^_hipA_. This is expected, as a lower number of CPG—acting on proximal muscles—will have a lower frequency-driven entrainment capacity.

Concerning the pathways acting on distal muscles (i.e., the 3FBL^osc^_ankle_ model), large changes in speed and step length are observed. However, contrary to what might be expected, an increase in frequency produces a decrease in speed. This is an artifact only possible because of the synchronization mechanism used to ensure the lock-in of the CPG with the mechanical system (see section 2.5.2.2). This effect is thus mainly related to a change in the duration of the burst of the feedforward signal (induced by the change in frequency), rather than to an entrainment between the two systems (i.e., CPG and musculoskeletal system). In other words, the observed gait modulations are due to a modulation of the shape of the signal (change in amplitude and/or duration).

Importantly, increases in speed induced by supraspinal influences on the different 3FBL models do not have the same effect on the gait characteristics (i.e., stride length and step duration). Modulation of the 3FBL^osc^_hipA_ or 3FBL^osc^_hipB_ parameters induce very little change in stride length (<5%). This is explained by the fact those CPGs are active only during swing and modulate the swing speed, but do not impact the swing length (and hence the stride length). Conversely, an increase in speed in the 3FBL^osc^_ankle_ induces a significant increase in stride length, as increasing the propulsive force will increase the swing length and thereby the stride length. As previously mentioned, the opposite effect is observed for the 3FBL^osc^_biArt_ (i.e., a decrease in stride length).

In real humans, it is known that, up to a certain point, increases in speed are usually accomplished by a decrease in step duration (i.e., increase in frequency), as well as by an increase in stride length (Murray et al., 1966). As expected, the 4 models exhibit a decrease in step duration with the increase in speed. Interestingly, only a modulation occurring on distal muscles also shows an increase in stride length, suggesting the propulsive force modulation as a means of velocity control.

Results suggest 2 ways of controlling speeds: (1) frequency modulation of CPGs acting on proximal muscles, (2) modulation of burst duration, amplitude and timing of CPGs acting on distal muscles.

## 4. Discussion

### 4.1. FBL

The analysis of gaits generated by the optimized FBL model (see section 3.1 for details) highlighted several similarities to healthy humans. Moreover, some solutions of different runs from the same optimization process showed ANKLE kinematics similarities to children suffering from cerebral palsy, highlighting the role that the FBL model could play in terms of modeling locomotion diseases. Children with cerebral palsy show a typical ANKLE flexion (instead of extension) in the early stance, followed by a double bump, visible at both the angle and torque level (Iosa et al., 2010). This is conceivably linked to a reduced hip range of motion, a weakness of tibialis anterior and/or a hypertone of gastrocnemious. Suprisingly some of the solutions described in section 3.1, such as solution 10 (orange line in Figures 5B,D right), show both features observed in children with cerebral palsy, i.e., ANKLE flexion in early stance and the double bump visible in both the torque and the angle. Furthermore, solution 10 shows a smaller HIP range of motion compared to solution 1. Finally, the tibilias anterior was found less active at the beginning of gait cycle compared to human physiological gait, as reported for children with cerebral palsy. Conversely, the double bump noted in the model seemed not to be related to an increased muscular activity of gastrocnemious. These interesting similarities, as well as the potential role of the model in disease/injury modeling should be further investigated.

### 4.2. FBL extension

Our approach—to use a dynamical system model of CPGs playing the role of feedback predictors—offers an easy and intuitive way of studying the relative importance of the different feedback pathways, and allowed us to highlight several aspects regarding speed control.

#### 4.2.1. CPG modulations on both proximal and distal muscles allow speed control

Mixing a constant predictor (CPG-CST) and feedbacks for as little as one pathway already enables speed and step length control. Increasing the level of CPG-CST for one specific pathway results in a flattening of the original feedback signal. Flattening the “SOL←SOL MFF, ST” feedback (i.e., the SOL positive muscle force feedback, active during stance) induces a clear decrease in both the gait speed and stride length, while flattening the “HF←HF MLF, SW” feedback (i.e., the HF negative muscle length feedback, active during swing) induces a clear decrease in the gait speed, but has little effect on the stride length (see Figure 6B). Those two observations confirm the intuition that speed changes would arise differently, depending on whether the control is applied during stance or swing. While speed control arising from stance control would more likely use extensor distal muscles, a speed control arising from swing control would more likely use proximal muscles. On the one hand, to be effective, a control acting during the stance should affect the propulsive force, which is mainly controlled by extensor muscles acting on the ankle joint (i.e., SOL and GAS muscles). It is thus not surprising that a modulation of feedback pathways acting on ankle extensor muscles during the stance affects the speed of locomotion (see Figure 6A). The effect on stride length is understood as the result of the modulation of the propulsive force: decreasing the propulsive force will decrease the swing length and thereby decrease the stride length. On the other hand, for the control acting during the swing at the level of the HIP flexors, the decrease in speed is not accompanied with any clear reduction in stride length (see Figure 6B green), meaning that it is the speed of the swing, but not its amplitude that induced the change in speed.

Similarly, the 3FBL models with CPG components acting on different groups of muscles confirm that speed control can arise from distal muscles extensors during the stance phase, and proximal muscles during the swing phase. We show that changes in speed, induced by a modulation of feedforward signals acting at the level of the ankle muscles, is unlikely due to a modulation of the frequency of the CPG network (see section 3.3.2), but rather induced by changes in burst duration and timing. Conversely, the results from a control acting during the swing at the level of proximal muscles shows that they could, indeed, be due to a modulation of the frequency of a CPG network.

When the CPG activity is modulated, the rest of the system (i.e., the remaining feedbacks) should adapt to the new conditions. Therefore, it is the combined effects of both CPGs and feedbacks that changes the gait properties (such as speed, step length, step duration). It has already been demonstrated that feedbacks acting at the level of the ankle produce such speed-adaptive behaviors (Markowitz et al., 2011). Here we show that this is true regardless of whether the control is applied at the level of proximal or distal muscles.

The proposed spinal architecture was able to generate speed transition ranging from 0.75 to 1.35 [m/s]. While this can seem relatively small compared to the controller proposed in Song and Geyer (2012), in which speed transition ranging from 0.8 to 1.6 [m/s] were obtained, the strategy proposed in this article has the advantage that changes in speed can be obtained without changing the reflex parameters. Furthermore, as the proportion of feedbacks vs. CPGs (i.e., α vector) of the 3FBL models were hand tuned, larger range of speed could be obtained through optimization. Finally, co-optimizing the feedback and feedforward components could also increase the range of speed. Indeed, as already stated, the 3FBL can be viewed as a system made of two components: a feedforward component and a corrective term, accounting for the differences between the feedback and the feedforward pathways (see section 3.2.3). In this context, the FBL model is a 3FBL model where the feedforward component is zero: the feedback parameters of the FBL are thus optimized for a model without any feedforward component. In this regard, since the 3FBL models were designed on top of an existing FBL model, the feedback parameters are not optimized to work with a non-zero feedforward component. This could also explain the low robustness of the 3FBL^min^_fdb_ model. Furthermore, in a biological point of view, it is obvious that the feedforward components should evolve together with the feedback components. Consequently, in the future, we will investigate the co-evolution of the feedforward and feedback components.

#### 4.2.2. Stable locomotion is produced even with a significant decrease in feedback activity

The 3FBL^min^_fdb_ model shows that stable locomotion can be produced despite a significant decrease in feedback activity. Indeed, stable walking is produced even with a 65% percent reduction in muscle feedback activity. As expected, this large decrease in feedback activity reduces the robustness of the gait to external perturbations (pushes and slope variation), and also considerably reduces the possibilities to control the gait (change in speed/stride length are not possible). This shows that some pathways are more important than others regarding their role as gait stabilizer which can be beneficial to both perturbation resistance and control of the gait.

#### 4.2.3. Exploiting the low dimensional organization of feedback pathways

Interestingly, in all the optimized FBL gaits, all the feedback pathways can be represented with as little as 4 signals found by non-negative matrix factorization (98% correlations between the original signals and the reconstructed one, data not shown). Since motoneurons are a simple linear combination of feedback pathways, the same conclusions are valid when analyzing the motoneurons signals. This low dimensional representation is also found in humans EMG patterns (Clark et al., 2009; Dominici et al., 2011), where only 4 signals, the so-called “motor-primitives,” are necessary to faithfully represent the EMG patterns of adult human walking. It would thus be interesting to exploit this low dimensional structure when modeling the feedforward components. In other words, we could model the CPGs as a set of motor-primitives that can be combined together to generate the different motoneurons states. Therefore, instead of viewing the CPG as a feedback predictor, we would view it as a motoneuron predictor. Based on the presented results, our new hypothesis is that the modulation of the timing, amplitude and duration of the motor-primitive will offer a better control of the gait, in terms of speed, stride length, gait transition and adaptation to increasing/decreasing slope.

## 5. Conclusion

In this work, we presented a method to introduce CPGs as feedforward components in a feedback based model of human walking. The proposed strategy is based on the idea that, in a feedback driven system, the feedforward component can be viewed as a feedback predictor. We implemented the feedback predictors using morph oscillators as abstract models of biological CPGs. Thanks to the intrinsic robustness inherited from the feedback pathways, the modulation of CPGs network’s frequency and amplitudes were possible, over a broad range, without affecting the overall walking stability. Furthermore, the modulation of the CPGs network’s parameters allowed smooth and stable speed changes in a range of 0.6 [m/s]. Preliminary results shows that the same strategy can be used to adapt to larges increase in slope (up to 30%) and to broader speed range (up to 0.8 [m/s]) suggesting that the idea of using feedback predictor as gait modulator can be extended to a large range of applications, highlighting the role biological CPGs could play on top of a reflex based rhythmic movement.

### Conflict of interest statement

The authors declare that the research was conducted in the absence of any commercial or financial relationships that could be construed as a potential conflict of interest.

## Footnotes

^1^The NMM library can be found online at https://bitbucket.org/efx/libnmm)

^2^The Webots implementation of the NMM library can be found online at https://bitbucket.org/efx/sml
