# Supplementary material for: The contribution of a central pattern generator in a reflex-based neuromuscular model
Source: Front Hum Neurosci. 2014 Jun 26;8:371. doi: 10.3389/fnhum.2014.00371 (PMC4071613; doi:10.3389/fnhum.2014.00371)
Supplement: Supplementary file 1 [file DataSheet1.PDF]

Frontiers in Neuroscience

Research Article
May 28, 2014

The Contribution of a Central Pattern Generator in a Reflex-Based Neuromuscular Model

Florin Dzeladini [1,*], Jesse van den Kieboom [1] and Auke Ijspeert [1]

[1] *BioRob, STI IBI, EPFL, Lausanne , Switzerland*

Correspondence*:
Florin Dzeladini
Biorob, STI IBI, Station 14, INN 239, EPFL, Lausanne, 1015, Switzerland,
florin.dzeladini@epfl.ch

Neuro-motor control

SUPPLEMENTAL DATA

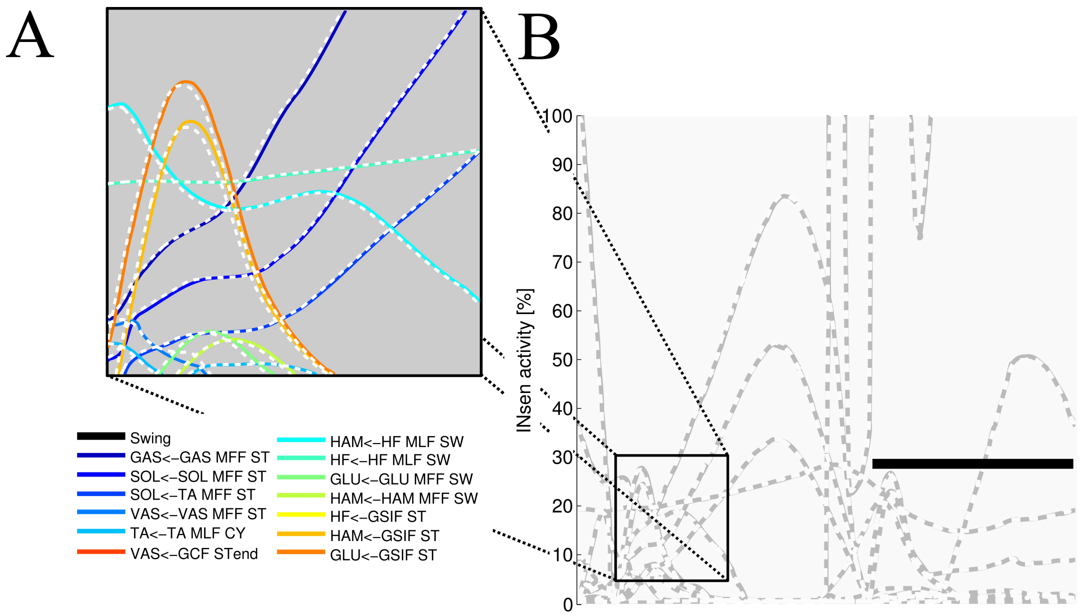

Supplementary Figure 1. Actual $\mathrm{IN}_{\mathrm{sen}}$ signals (dotted lines) and the reproduced signals (thick lines) for the the worst gait, in terms of $\mathrm{IN}_{\mathrm{sen}}$-$\mathrm{IN}_{\mathrm{cpg}}$ similarity. A) zoom in a subpart of the INsen activity. We clearly see that the errors between the reproduced signal and the real one are very small. B) INsen activity over one cycle. Note that we do not reproduce the “HF$\leftarrow$TLF SW” and the “VAS$\leftarrow$KNEE OPF” $\mathrm{IN}_{\mathrm{sen}}$, because their roles as feedback is clear: the first gives stability to the gait by generating larger steps when the body leans forward, and the second because it prevents knee overextension.

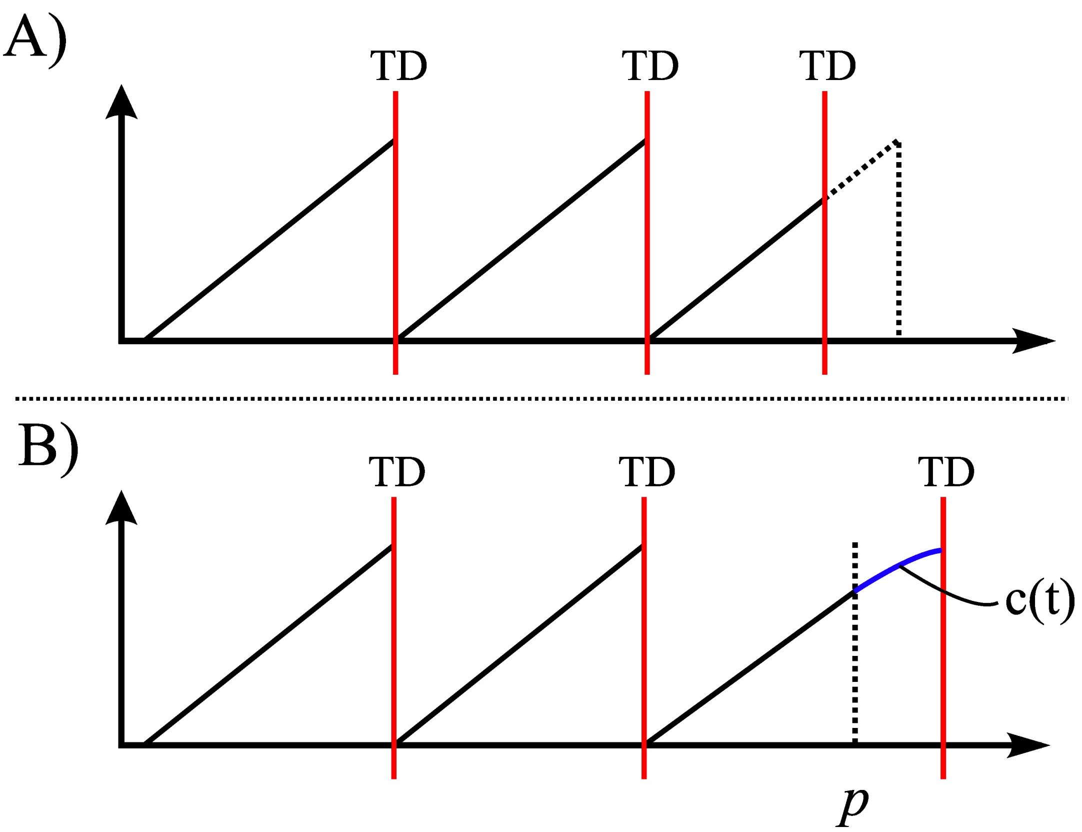

Supplementary Figure 2.CPG-OSC synchronization mechanism. A) If the central clock is to slow compared to the walking (i.e. the touchdown/takeoff event occurs before the oscillator has finished its period) the phase is simply reset. B) If the central clock is to fast compared to the walking, a slowing down mechanism enters in action. The mechanism enters in action at a defined percentage of the period ($p = 90\%$), ensuring that the oscillator will not finish its period before the synchronization event (SE) occurs.

REFERENCES

Geyer, H. and Herr, H. (2010), A muscle-reflex model that encodes principles of legged mechanics produces human walking dynamics and muscle activities, *IEEE Transactions on Neural Systems and Rehabilitation Engineering*, 18, 3, 263–273, doi:10.1109/TNSRE.2010.2047592

Geyer, H., Seyfarth, A., and Blickhan, R. (2003), Positive force feedback in bouncing gaits?, *Proceedings of the Royal Society B: Biological Sciences*, 270, 1529, 2173–2183, doi:10.1098/rspb.2003.2454

Winter, D. A. (2009), Biomechanics and Motor Control of Human Movement (John Wiley & Sons, Inc., Hoboken, NJ, USA)

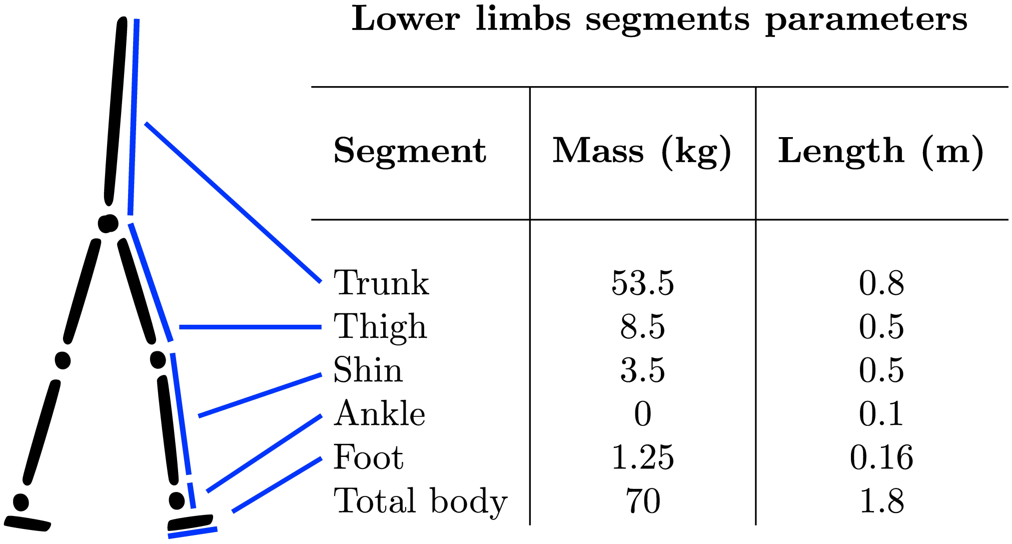

Lower limbs segments parameters

Segment	Mass (kg)	Length (m)
Trunk	53.5	0.8
Thigh	8.5	0.5
Shin	3.5	0.5
Ankle	0	0.1
Foot	1.25	0.16
Total body	70	1.8

Supplementary Figure 3. Segments weight and length distribution based on anthropometric data from **Winter** (2009).

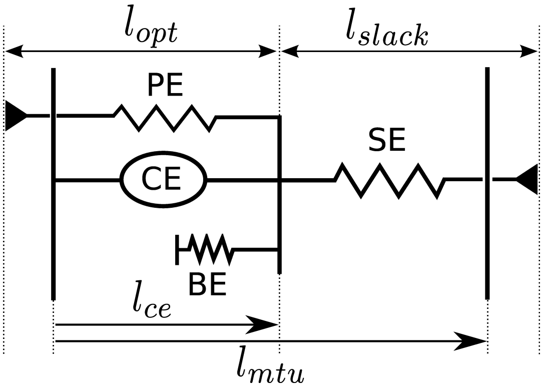

Supplementary Figure 4. Schematic view of a muscle tendon unit (MTU) adapted from **Geyer and Herr** (2010). In normal walking condition (no overextension nor overflexion), only the serial element (SE) and the contractile element (CE) are active. Two other passive elements are added in parallel of CE: BE that engages if tendon is slack (i.e if $l_{mtu} - l_{\text{CE}} = l_{\text{SE}} < l_{slack}$), preventing muscle collapse, and PE, that engages when the muscle stretches beyond its optimal length (i.e if $l_{\text{CE}} > l_{opt}$), preventing the muscle to extend beyond a certain length.

Supplementary Table 1. FBL model parameters list and their respective range. The parameters are tuned by optimization. ks_* and δ_{ref} are the stability feedback related parameters. wf_* are muscle force feedback gains, wl_* muscle length feedback gains and $l*$ muscle length offsets. s^0* are the muscle basal activities. $k_{\delta knee}$ is the gain of the knee overextension prevention feedback. ΔS is a constant term added to the HF and substracted from the GLU during stance.

Name	Range	Name	Range
wf_{sol}	$[\,0.8; 1.6\,]$	ks_{lean}	$[\,0.0; 2.0\,]$
wf_{ta_sol}	$[\,0.1; 0.8\,]$	ks_{bw}	$[\,0.8; 1.4\,]$
wf_{gas}	$[\,0.3; 1.6\,]$	ks_{p1}	$[\,0.8; 1.4\,]$
wf_{vas}	$[\,0.9; 1.8\,]$	ks_{p2}	$[\,0.5; 1.4\,]$
wf_{ham}	$[\,0.2; 1.0\,]$	ks_d	$[\,0.8; 1.4\,]$
wf_{glu}	$[\,0.2; 0.9\,]$	s^0_{sol}	$[\,0.01; 0.1\,]$
wl_{ta}	$[\,1.0; 3.0\,]$	s^0_{ta}	$[\,0.01; 0.1\,]$
wl_{hf}	$[\,0.2; 1.5\,]$	s^0_{gas}	$[\,0.01; 0.1\,]$
wl_{ham}	$[\,0.0; 3.0\,]$	s^0_{vas}	$[\,0.01; 0.1\,]$
l^{ta}_{offset}	$[\,0.0; 1.0\,]$	s^0_{ham}	$[\,0.01; 0.1\,]$
l^{hf}_{offset}	$[\,0.2; 1.0\,]$	s^0_{glu}	$[\,0.01; 0.1\,]$
l^{ham}_{offset}	$[\,0.7; 1.0\,]$	s^0_{hf}	$[\,0.01; 0.1\,]$
		$k_{\delta knee}$	$[\,0.0; 3.0\,]$
		ΔS	$[\,0.0; 1.05\,]$

Supplementary Table 2. Summary of solution rank in terms of CoT and number of $\mathrm{IN_{sen}}$ that could not be replaced by a $\mathrm{IN_{cpg}}$ model while keeping over pathway purely feedback (i.e. $\alpha = 1$). The first column gives the solution rank in terms of CoT, the second column shows the number of $\mathrm{IN_{sen}}$ that could not be replaced by a $\mathrm{IN_{cpg}^{osc}}$ model, the third column shows the number of $\mathrm{IN_{sen}}$ that could not be replaced by $\mathrm{IN_{cpg}^{cst}}$ model. Note that the VAS←KNEE OPF and "HF←TLF SW" were not considered, thereby limiting the number of effective $\mathrm{IN_{cpg}}$ to 13.

CoT	$\mathbf{IN_{cpg}^{osc}}$	$\mathbf{IN_{cpg}^{cst}}$
2	1/13	7/13
3	1/13	7/13
6	1/13	7/13
8	1/13	8/13
4	2/13	7/13
1	3/13	8/13
10	3/13	8/13
9	5/13	7/13
5	5/13	8/13
7	6/13	6/13

Supplementary Table 3. Summary of the main vectors / matrices used in the control loop. The model uses a total of 26 sensors (9 muscle sensors, 1 knee joint angle sensors and 2 ground sensors per limb plus 2 trunk sensors). Each sensory interneurons receives connection from one sensor (except the stability sensory interneuron that receives input from the trunk angle and ground reaction forces). The number of sensory interneurons is of 15 per limb see Table **??** for details. The number of effective CPG is 9 for the 3FBL models (only the muscle feedbacks are considered) and 13 otherwise (muscle feedbacks + stability feedbacks considered).

Vector / matrices summary

	**Dim**	**Description**
X_{sen}	26x1	Vector of sensors states
W	30x26	Connection weight between sensors and sensory interneurons
$X_{\mathrm{in_{sen}}}$	30x1	Vector of sensory interneurons states
$X_{\mathrm{in_{cpg}}}$	30x1	Vector of CPG interneurons states
G^s	14x30	State machine matrix wiring interneurons to motoneurons
X_{mn}^0	14x1	Vector a basal motoneuron activities
X_{mn}	14x1	Vector of all motoneurons states
X^{MTU}	14x1	Vector of all MTU stimulation
A	14x1	Vector of all MTU activitation level

Supplementary Table 4. The 3 stages used during optimization of the FBL. All evaluated criteria are maximized. d is the distance covered by the simulated biped, d_{lim} the maximum distance (simulation stops when $d \geqslant d_{\text{lim}}$), v is the mean speed, v_{opt} the desired mean speed, E is the energy expenditure, P is a penalty term accounting for knee overextension (see **Geyer et al.** (2003) for details).

**stage**	**fitness fct**	**end condition**
1	d	$d >= d_{\text{lim}}$
2	$\lvert v - v_{opt} \rvert$	$\lvert v - v_{opt} \rvert < 0.05$
3	$-P$	$P < 0.01$
4	$-E$	

Supplementary Table 5. Range of joints angle outside of which soft limit engages. The soft limit models the action of ligaments to work against unnatural movement, thus preventing injuries **Geyer and Herr** (2010).

**Joint**	$\theta_{\mathbf{min}}$	$\theta_{\mathbf{max}}$
HIP	20	230
KNEE	45	175
ANKLE	70	130
